# Repurposing CRISPR/Cas to Discover SARS‐CoV‐2 Detecting and Neutralizing Aptamers

**DOI:** 10.1002/advs.202300656

**Published:** 2023-05-19

**Authors:** Ju Zhang, Airu Zhu, Miao Mei, Jing Qu, Yalan Huang, Yongshi Shi, Meiying Xue, Jingfang Zhang, Renli Zhang, Bing Zhou, Xu Tan, Jincun Zhao, Yu Wang

**Affiliations:** ^1^ State Key Laboratory of Stem Cell and Reproductive Biology Institute of Zoology Chinese Academy of Sciences Beijing 100101 China; ^2^ University of Chinese Academy of Sciences Beijing 100049 China; ^3^ Beijing Institute for Stem Cell and Regenerative Medicine Beijing 100005 China; ^4^ College of Life Sciences and Oceanography Shenzhen University Shenzhen 518060 China; ^5^ State Key Laboratory of Respiratory Disease National Clinical Research Center for Respiratory Disease Guangzhou Institute of Respiratory Health the First Affiliated Hospital of Guangzhou Medical University Guangzhou 510120 China; ^6^ Tsinghua‐Peking Center for Life Sciences Beijing Advanced Innovation Center for Structural Biology Beijing Frontier Research Center for Biological Structure MOE Key Laboratory of Bioorganic Phosphorus Chemistry & Chemical Biology School of Pharmaceutical Sciences Center for infectious Disease Research School of Medicine Tsinghua University Beijing 100084 China; ^7^ Institute of Pathogenic Organisms Shenzhen Center for Disease Control and Prevention Shenzhen 518055 China; ^8^ School of Life Sciences Beijing University of Chinese Medicine Beijing 100105 China

**Keywords:** CRISmers, CRISPR/Cas, RNA aptamers, SARS‐CoV‐2

## Abstract

RNA aptamers provide useful biological probes and therapeutic agents. New methodologies to screen RNA aptamers will be valuable by complementing the traditional Systematic Evolution of Ligands by Exponential Enrichment (SELEX). Meanwhile, repurposing clustered regularly interspaced short palindromic repeats (CRISPR)/CRISPR associated systems (Cas) has expanded their utility far beyond their native nuclease function. Here, CRISmers, a CRISPR/Cas‐based novel screening system for RNA aptamers based on binding to a chosen protein of interest in a cellular context, is presented. Using CRISmers, aptamers are identified specifically targeting the receptor binding domain (RBD) of the spike glycoprotein of severe acute respiratory syndrome coronavirus 2 (SARS‐CoV‐2). Two aptamer leads enable sensitive detection and potent neutralization of SARS‐CoV‐2 Delta and Omicron variants in vitro. Intranasal administration of one aptamer, further modified with 2’‐fluoro pyrimidines (2’‐F), 2’‐*O*‐methyl purines (2’‐O), and conjugation with both cholesterol and polyethylene glycol of 40 kDa (PEG40K), achieves effective prophylactic and therapeutic antiviral activity against live Omicron BA.2 variants in vivo. The study concludes by demonstrating the robustness, consistency, and potential broad utility of CRISmers using two newly identified aptamers but switching CRISPR, selection marker, and host species.

## Graphical abstract

CRISmers, a novel system for screening RNA aptamers, provides an endogenous biological context for the folding of aptamersand proteins, and their interaction. Performance of CRISmers is showcased by identification of a potent neutralizing aptamer against the live severe acute respiratory syndrome coronavirus 2 (SARS‐CoV‐2) Omicron variant in vivo. Further, CRISmers is compatible with varying systems, host species, and cellular enrichment methods.

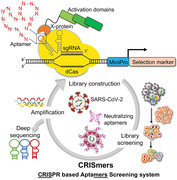

## 1 Introduction

Nucleic acid aptamers are a class of short, single‐stranded DNA (ssDNA) or RNA oligonucleotides that bind to a wide range of targets, from proteins to whole cells, with high specificity and affinity. RNA aptamers have higher conformational flexibility than their DNA counterparts.^[^ ^1^ ^]^ Compared with antibodies and peptides, aptamers exhibit several advantages, including ease of synthesis and storage, minimal batch‐to‐batch variation, cell‐ or animal‐free composition, smaller size and shorter circulation, a wider range of targets, lower immunogenicity, the availability of site‐specific chemical modifications, and bio‐conjunction.^[^ ^2^ ^]^ Based on their unique biological and chemical characteristics, aptamers have been screened and developed for applications in diagnostics and therapeutics for many anomalies such as oculopathy,^[^ ^3^ ^]^ inflammation,^[^ ^4^ ^]^ and cancer.^[^ ^5^ ^]^ The latest developments utilizing aptamers' capability to target a protein or cell type to enhance the selectivity of delivery for another moiety, such as a chemical drug Aptamer‐Drug Conjugates or Aptamer Lipid‐NanoParticles, might lead to a new paradigm shift in the field.^[^ ^6^, ^7^, ^8^ ^]^

RNA aptamers and their screening methodology, SELEX, were first reported in 1990.^[^ ^9^, ^10^, ^11^ ^]^ SELEX represents an experimental directed evolution system to enrich for RNA aptamers with a certain property. In general, one cycle of SELEX includes the following steps: construction of a nucleotide sequence library, acquisition of the target molecules, incubation, affinity selection, and recovery of binding complexes, amplification and analysis of recovered nucleotide sequences. SELEX has long been the gold standard for screening ssDNA or RNA aptamers, providing a large number of high‐quality modalities including several being approved or examined in clinic.^[^ ^1^, ^12^ ^]^ A SELEX library typically consists of nucleotide sequences with a central region of random sequence, typically 20–60 nucleotides (nt) in length to present sufficient sequence diversity, and two flanking fixed sequences, typically 15–25 nucleotides in length, sufficient to be specifically complemented with primers for Polymerase Chain Reaction (PCR) amplification.^[^ ^1^, ^13^, ^14^ ^]^ Nowadays, SELEX routinely utilizes next‐generation sequencing to efficiently analyze the enriched oligonucleotides from a large amount of data. Modified SELEX, such as Microfluidic‐SELEX has been developed, which involves the use of microfluidic chips that allow for control of fluid movement and mixing.^[^ ^15^ ^]^ SELEX has been carried out against purified proteins, whole cells, and live animals. Nonetheless, inherent limitations of SELEX suggest the need to develop complementary methodologies. For instance, recombinant‐protein‐based SELEX may not recapitulate the native conformations of target proteins.^[^ ^16^ ^]^ Cell‐based SELEX screening can only efficiently target proteins on the cell surface, which limits the number of applicable proteins and/or the concentration and conformation of the target proteins.^[^ ^16^, ^17^ ^]^ Animal‐based SELEX screening process is costly with a lack of specificity and throughput.^[^ ^2^ ^]^ Therefore, complementary screening systems that are compatible with a native biological context but are also efficient and scalable are highly desirable for the field.

CRISPR /Cassystem, RNA‐guided endonucleases that cleave double‐strands of DNA, has proven to be a powerful and versatile genome editing tool.^[^ ^18^, ^19^ ^]^ Reengineering and repurposing of CRISPR/Cas have enabled applications far beyond its native endonuclease function, including base editing and prime editing without the double‐stranded break,^[^ ^18^ ^]^ transcriptional activation and interference (CRISPRa and CRISPRi, respectively), epigenome engineering, and genomic DNA labeling.^[^ ^20^ ^]^ This bewilderingly wide range of applications of the CRISPR/Cas system owes to its high specificity, facile programmability, and great compatibility with different cells, tissues, and species.

Since the outbreak of the Coronavirus Disease 2019 (COVID‐19), scientists worldwide are spearheading in all possible directions simultaneously with a common goal toward drug and vaccine development against the virus. Hereof, nucleic acid medications represented by the mRNA vaccines have gained enormous momentum,^[^ ^21^, ^22^ ^]^ while other forms, including siRNAs and aptamers, are also attracting significant interest.^[^ ^23^ ^]^ Aptamers bear exciting opportunities in the development of diagnostics and therapeutics against SARS‐CoV‐2, given their beneficial properties distinct from those of antibodies and peptides. Several DNA and RNA aptamers screened by traditional SELEX have been reported for the detection and inhibition of SARS‐CoV‐2 in vitro.^[^ ^24^, ^25^, ^26^, ^27^, ^28^, ^29^, ^30^, ^31^, ^32^, ^33^, ^34^, ^35^, ^36^, ^37^, ^38^, ^39^ ^]^

Several natural aptamers, such as bacteriophage bacMS2 and PP7 and their binding proteins, have been utilized in engineering CRISPR/Cas systems for various applications, such as transcriptional activators and repressors, epigenetic modifiers, and fluorescent genomic DNA probes.^[^ ^40^, ^41^ ^]^ In addition, an aptamer inhibitor against Cas9 protein was identified by SELEX to control its activity.^[^ ^42^ ^]^ And a series of aptamer‐linked CRISPR‐based biosensors have been designed for biomarkers and pathogen detection.^[^ ^43^, ^44^ ^]^ However, to the best of our knowledge, CRISPR/Cas systems have not yet been repurposed for screening aptamers so far. Here, we present CRISmers, a novel RNA aptamer screening platform based on a new strategy to repurpose CRISPR/Cas. CRISmers brings aptamer screening system to the intracellular native biological context, thus making it less vulnerable to environmental fluctuation and providing a more biologically relevant condition for RNA and protein folding and their interaction. In CRISmers, individual cells as physically distinct units are used to distinguish functional binding events from background noise. This might greatly increase the specificity and robustness of the system by avoiding the use of naked nucleotides, remnants of which could be easily amplified during PCR and bias the sequencing pool in SELEX. Using CRISmers, we successfully identified RNA aptamers with high specificity and affinity for the RBD of SARS‐CoV‐2 spike glycoprotein. Strikingly, an aptamer lead showed its potential as a new antiviral modality with potent neutralizing activity against live SARS‐CoV‐2 of the Omicron BA.2 variants both in cell culture and in animal experiments. The potential broad utility and robust and consistent performance of CRISmers were also demonstrated by using orthogonal CRISPR/Cas systems, selection markers, and host species. Therefore, while the very first CRISmers screen targeted SARS‐CoV‐2, it can play an important role in the post‐COVID‐19 era in many directions as a fundamental tool to identify novel aptamers and RNA‐protein interactions in general.

## 2 Results

### 2.1 Conceptual Design of CRISPR/Cas9‐Based Aptamer Screening System

**Figure 1:**
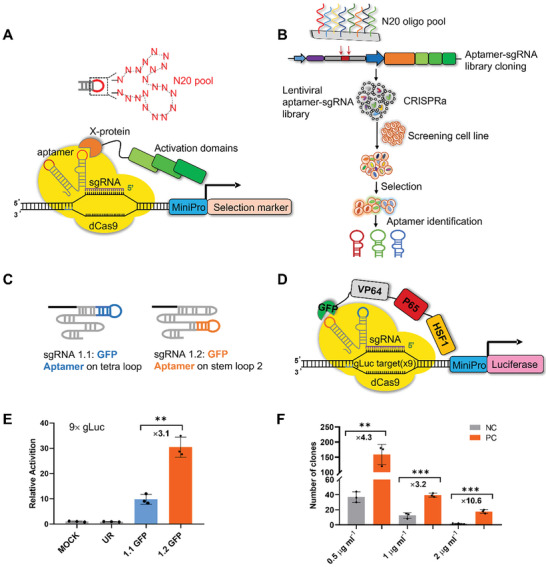
Conceptual design, proof of concept, and key parameter characterizations of a CRISPR/Cas9‐based aptamer screening system. A,B) Schematic illustrations of a conceptual molecular device repurposing CRISPR/Cas9 system to screen RNA aptamers and the envisioned screening process. Key components are color‐coded as the following: the aptamer is colored in red; the POI is colored in orange; the activation domains are colored in green; the mini promoter is colored in blue; the selection marker is colored in pink. C) a GFP aptamer was incorporated in the tetraloop of sgRNA scaffold (sgRNA 1.1) and the stem loop2 of sgRNA scaffold (sgRNA 1.2), respectively. D) Illustration of the luciferase reporter assay to detect the affinity binding of an aptamer to its GFP target. E) Results of a luciferase assay shown in Figure 1D, using 9 repeats of gLuc sgRNA target sequences (9 × gLuc) to amplify the sensitivity. F) Quantification of the number of clones survived upon lentiviral delivery of sgRNA 1.2 and GFP‐VPH into No.4‐53 Monoclonal dCas9/9 × gLuc‐Puro cell line (Figure S2, Supporting Information). The Positive Control/Negative Control (PC/NC) ratios were shown on the top and the puromycin concentration used was indicated at the bottom. Mock: transient transfection of control plasmids. UR: gLuc sgRNA 1.2‐the negative control sgRNA used in luciferase reporter assay was gLuc target without any aptamer appendage (the sgRNA‐1.2 BsmBI scaffold). 1.1 GFP: gLuc sgRNA 1.1‐GFP aptamer. 1.2 GFP: gLuc sgRNA 1.2‐GFP aptamer. NC: Lentiviral‐sgRNA 1.2‐the negative control sgRNA used in luciferase reporter assay was gLuc target without any aptamer appendage (the sgRNA‐1.2 BsmBI scaffold)‐GFP‐VPH. PC: Lentiviral‐sgRNA 1.2‐GFP apt‐GFP‐VPH. *n* = 3 biological replicates. ^**^ *p* < 0.01, ^***^ *p* < 0.001, two‐tailed *t*‐tests. GraphPad Prism 8.0.1 software was used for statistical analysis.

Based upon our previous experience in CRISPRa,^[^ ^45^ ^]^ we envisioned a design wherein an aptamer is cloned into a loop on the single guide RNA (sgRNA) scaffold while the target Protein of Interest (POI) is fused with transcriptional transactivation domains. Only when an aptamer binds to the POI can the transactivation domains be recruited to the nuclease‐deactivated Cas9 (dCas9)‐sgRNA complex and docked to the sgRNA‐target sequence upstream of a minipromoter, which drives the expression of a selection marker gene (**Figure** **1**A). The selection pressure is afforded by adding the selection antibiotic to enrich for cells expressing sgRNA scaffolds containing aptamers that bind the POI. Such a sgRNA library could be easily obtained by oligo synthesis containing random sequences, followed by pooled ligation into a lentiviral sgRNA expression construct. Pooled sgRNA constructs can be packaged into lentivirus particles and used for cellular transduction so that aptamer‐encoding DNA sequences are integrated into the genomes of host cells. Upon selection with the antibiotics, aptamers with affinity for the POI can be identified by Next‐Generation Sequencing (NGS) of the genomic DNA of the surviving cells (Figure 1B).

### 2.2 Validation and Characterization of CRISPR/Cas9‐Based RNA Aptamer Identification

We first tested our conceptual design by using a Green Fluorescent Protein (GFP) aptamer identified previously from SELEX,^[^ ^46^ ^]^ and a firefly‐luciferase reporter, in which reporter expression could be activated by recruitment of CRISPRa to the upstream sgRNA target sequence derived from gaussia luciferase (gLuc target).^[^ ^45^ ^]^ The GFP aptamer was appended to the sgRNA tetra loop (sgRNA 1.1) or stem loop 2 (sgRNA 1.2), respectively (Figure 1C), and GFP was fused with VP64, P65, and HSF1 transactivation domains (GFP‐VPH) (Figure 1D). A monoclonal Human Embryonic Kidney 293T (HEK293T) cell line stably expressing dCas (293T/dCas) was co‐transfected with plasmids expressing sgRNA 1.1‐GFP apt or sgRNA 1.2‐GFP apt, GFP‐VPH, and luciferase reporter (Figure 1E). We detected a significant luciferase signal when its transcription was driven by 9 tandem repeats, but not a single copy, of gLuc sgRNA target sequences, indicating amplified assay sensitivity by recruiting more CRISPRa devices (Figure 1E; Figure S1, Supporting Information). Notably, GFP aptamer appended to stem loop 2 (sgRNA 1.2) showed more efficient activation than that appended to the tetraloop (sgRNA 1.1) (Figure 1E).

Next, we replaced the luciferase reporter with a puromycin (Puro) selection marker. Monoclonal dCas9/9 × gLuc‐Puro cell lines were generated by sequential lentiviral delivery of dCas9 and 9 × gLuc‐Puro reporter. These lines were screened upon a third round of lentiviral infection delivering a construct co‐expressing the gLuc sgRNA with GFP aptamer fused to stem loop 2 (sgRNA 1.2) and the GFP‐VPH fusion protein (Positive Controls, PC), while a sgRNA with insertion of a random sequence in place of the GFP aptamer served as Negative Controls (NC) (Figure 1F; Figure S2A, Supporting Information). Puromycin was applied at different concentrations of 0.5, 1, and 2 µg mL^−1^, representing escalating selection pressure. Surviving clones were quantitated for each dCas9/9 × gLuc‐Puro cell line (Figure S2B, Supporting Information). Generally, the highest enrichment ratios (PC/NC) were observed with 2 µg mL^−1^ puromycin, an indication of proper selection pressure. Notably, clone No.4‐53 showed consistently high PC/NC ratios and was chosen for the following screening in this study (Figure 1F).

### 2.3 A proposed Work‐Flow of the CRISmers System

**Figure 2:**
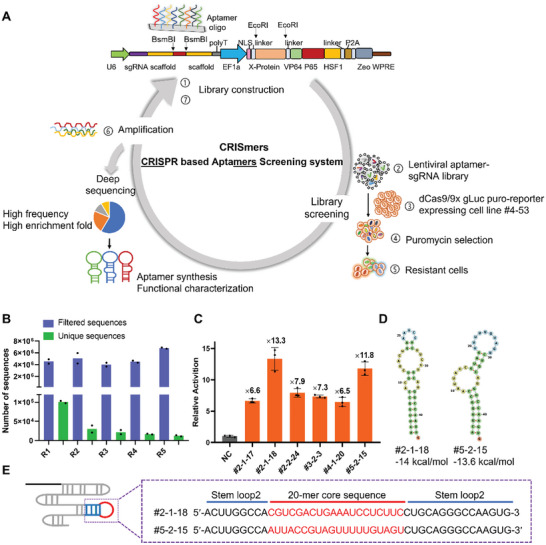
A proposed work‐flow of the CRISmers system and CRISmers screening hits. A) Schematic illustration of the CRISmers: RNA aptamer screening system based on CRISPR/Cas. The process of one screening cycle can be divided into these steps: 1) construction of a lentiviral‐CRISmers random library, 2) production of the the CRISmers random library lentivirus, 3) CRISmes random library lentivirus infection of No.4‐53 monoclonal dCas9 × gLuc Puro cell line, 4) selection for puromycin resistance cells, 5) preparation of genomic DNA from resistant cells, 6) selective PCR amplification of aptamer, deep sequencing, and analysis, 7) sub‐library construction if necessary. B) Deep sequencing analyses of harvested aptamer sequences after each round of CRISmers. R: Selection rounds. C) Results of a luciferase reporter assay (Figure 1D) for parallel comparison of aptamer hits showing the highest activity from the secondary screen (Figure S3 and Table S1, Supporting Information). NC: the negative control sgRNA used in luciferase reporter assay was gLuc target without any aptamer appendage (the sgRNA‐1.2 BsmBI scaffold). *n* = 3 biological replicates. D) The secondary structures and the free energy of the two aptamers predicted by Mfold webserver are shown. E) Illustration of the sequences of two aptamers (#2‐1‐18 and #5‐2‐15) showing the highest fold of activation in the luciferase assay in Figure 2C. The 20‐mer core sequence is colored in red (Table S1, Supporting Information).

Based on the working model and key parameters that we established from the proof‐of‐concept experiments described above, we proposed a CRISPR‐based RNA aptamer screening system (CRISmers) (**Figure** **2**A). The work‐flow of one screening cycle can be divided into the following steps: first, construct a lentiviral‐CRISmers random library. A pool of oligos containing a 20 nt random region flanked by two 40 nt fixed primer regions is synthesized and cloned into the stem loop 2 of gLuc sgRNA scaffold in a lentiviral expression construct. Restriction sites are in place for customization. Second, produce the CRISmers random library lentivirus. A pool of constructs co‐expressing the gLuc sgRNA‐aptamer library and one specific target protein of interest is packaged into lentivirus particles. Third, transduce the CRISmers random library lentivirus. The lentivirus particles are used to infect the No.4‐53 monoclonal dCas/9 × gLuc‐Puro cell line. Fourth, select for puromycin resistance. Puromycin (2 µg mL^−1^) is applied to the cell line to select for puromycin resistant cells. Fifth, extract genomic DNA. Sixth, read the screening information. Aptamer sequences are selectively amplified and analyzed by deep sequencing. Seventh, further CRISmers screening. The next cycle, when necessary, is initiated by subcloning the amplified aptamer sequences from the previous round into the original library shuttle construct. Primary hits identified from the screening are then further validated using a luciferase reporter assay.

### 2.4 RNA Aptamer Screening against the RBD of SARS‐CoV‐2 Spike Protein

Considering the urgent need to develop diagnostics and therapies against SARS‐CoV‐2 during the COVID‐19 pandemic, we targeted the RBD of SARS‐CoV‐2 spike protein as the POI of our first CRISmers screen. Two independent parallel screens with five rounds of each were conducted. In the first round, the pooled N20 lentiviral library of gLuc sgRNA appended with random 20 nucleotides (N20), a length sufficient to form configuration for effective protein binding,^[^ ^1^, ^13^, ^14^ ^]^ to its stem loop 2 was transduced at a Multiplicity of Infection (MOI) of ≈3 to maximize the representation of the aptamer population in the infected cells. Then the following rounds were performed at a lower MOI of ≈0.1–0.2 to increase the probability that most cells receive only one aptamer. Aptamer yield converged to lesser unique sequences in each following round (R), after filtering out non‐specific sequences (Figure 2B).^[^ ^47^ ^]^ In rounds 4 and 5 (R4 and R5), the number of unique sequences appears to be no longer reduced significantly, indicating that enrichment was saturated.

### 2.5 Secondary Screen and Validation of Top Hits

We calculated and ranked the top 0.1% high frequency sequences and the top 15 sequences with the highest fold‐of‐enrichment (ratio of copy numbers in comparison against the previous round of screening)^[^ ^47^ ^]^ from R2, R3, R4, and R5 pools and tested them in the aforementioned luciferase reporter assay as a secondary screen to validate the identified aptamers (Figure 1D; Figure S3, Supporting Information). It should be noted that the highly represented sequences may be biased products of PCR amplification, thus not necessarily exhibiting a high enrichment fold.^[^ ^47^ ^]^ Moreover, in R4 screening and especially in R5, some high‐frequency or highly enriched sequences have already been selected and verified in the previous rounds, an indication of sufficient enrichment and CRISmers’ performance consistency.

Aptamers that showed the most significant signal in the above secondary luciferase screening were identified and listed in Table S1 (Supporting Information). Among them, #2‐1‐18 and #5‐2‐15 showed the highest activity in a head‐to‐head comparison (Figure 2C,E). Therefore, we focused on these two aptamer leads hereafter. Figure 2D shows their secondary structures and free energy values predicted by Mfold webserver.^[^ ^48^ ^]^ Paralleled examination of previously reported RNA aptamers and DNA aptamers targeting SARS‐CoV‐2 Spike‐RBD in the luciferase reporter assay produced comparable levels of activation (Figure S4A, Supporting Information).^[^ ^24^, ^25^, ^26^, ^28^, ^30^, ^33^ ^]^

### 2.6 Using Aptamers to Detect SARS‐CoV‐2 Pseudovirus and Its Variants

**Figure 3:**
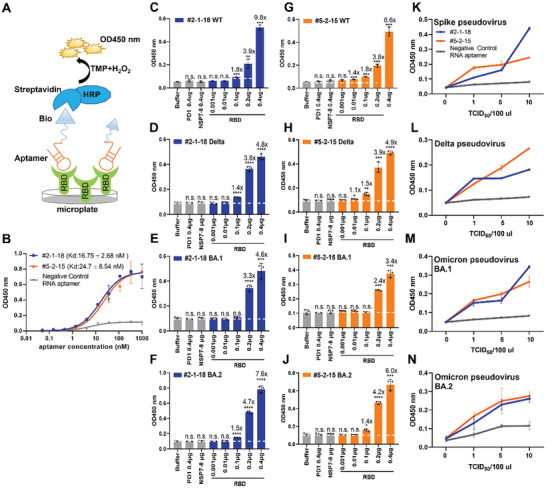
Examination of aptamer leads for detecting SARS‐CoV‐2 and its variants. A) A schematic depicting the enzyme‐linked oligonucleotide assay (ELONA) assay. B) Examination of aptamers for their dose dependent ELONA activity to a fixed concentration of 250 ng of the RBD of SARS‐CoV‐2 Spike protein. A negative control RNA aptamer was used as a negative control. Examination of binding activity to RBD recombinant protein of the original SARS‐CoV‐2 (C and G), Delta (D and H), Omicron BA.1 (E and I), and Omicron BA.2 (F and J) using 100 nM aptamers. Recombinant protein PD‐1 extracellular domain and SARS‐CoV‐2 NSP7‐8 complex were used as negative controls for specificity. Examination of binding activity to the original SARS‐CoV‐2 pseudovirus (K), Delta (L), and Omicron BA.1 and BA.2 (M,N) using 100 nM aptamers. A negative control RNA aptamer was used as a negative control for specificity. TCID50: 50% tissue culture infective dose. Data show mean ± SD. *n* = 3 biological replicates. ^*^ *p* < 0.05, ^**^ *p* < 0.01, ^***^ *p* < 0.001, ^****^ *p* < 0.0001, two‐tailed *t*‐tests. n.s., no significant difference. GraphPad Prism 8.0.1 software was used for statistical analysis.

We next measured the binding affinity of the two aptamers via an Enzyme‐Linked OligoNucleotide Assay (ELONA) (**Figure** **3**A).^[^ ^49^ ^]^ Both aptamers exhibited high‐affinity binding to recombinant RBD, with *Kd* values of 16.75 ± 2.68 and 24.7 ± 8.54 nM for aptamers #2‐1‐18 and #5‐2‐15, respectively (Figure 3B). We then examined the binding activity when using 100 nM aptamers in ELONA by titrating RBD recombinant proteins of the original SARS‐CoV‐2 (Figure 3C,G), Delta (Figure 3D,H), or Omicron BA.1 (Figure 3E,I), and BA.2 (Figure 3F,J), and SARS‐CoV‐2 particles pseudotyped with spike proteins of the original SARS‐CoV‐2 (Figure 3K), Delta (Figure 3L), or Omicron BA.1 and BA.2 variants (Figure 3M,N). Recombinant Programed Death 1 (PD1) extracellular domain, Non‐Structural Proteins 7 and 8 (NSP7‐8) protein complex of SARS‐CoV‐2, and a negative control RNA aptamer were included as negative controls in these assays. Both aptamers at 100 nM showed specific binding activity with RBD recombinant protein (Figure 3C,G) and all three pseudoviruses at a titer of 1 TCID_50_ (50% tissue culture infective dose) and higher (Figure 3K–N). Importantly, their binding activities were maintained at 4, 25, and 37 °C, indicating potential applications in a wide range of working temperatures (Figure S4B, Supporting Information).

### 2.7 Neutralizing Activity of Aptamers in Cell Culture against Live SARS‐CoV‐2 Variants of Concern

**Figure 4:**
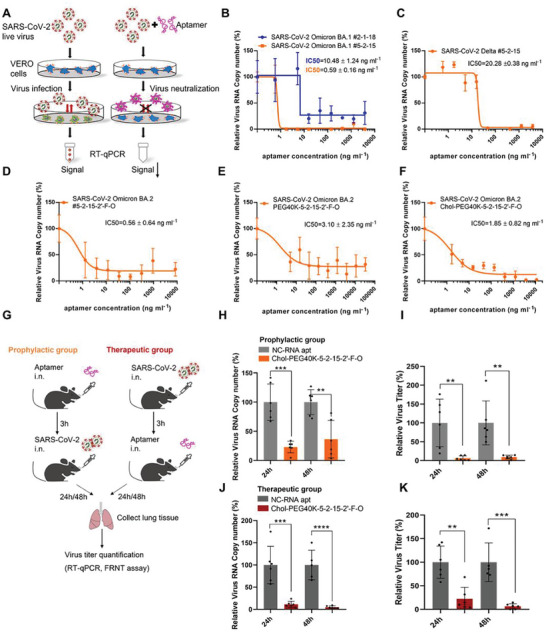
Neutralizing activity against live SARS‐CoV‐2 variants of concern. A) A cartoon showing the working principle of SARS‐CoV‐2 live virus neuralization assay. B) Quantification of relative virus RNA copy number to assess aptamer #2‐1‐18 and aptamer #5‐2‐15 for their activity against the infectivity of SARS‐CoV‐2 Omicron BA.1 variant. *n* = 3 biological replicates. C) Quantification of relative virus RNA copy number to assess aptamer #5‐2‐15 for its activity against the infectivity of SARS‐CoV‐2 Delta variant. *n* = 3 biological replicates. D) Quantification of relative virus RNA copy number to examine aptamer #5‐2‐15 with 2’‐F‐O modifications (#5‐2‐15‐2’‐F‐O) for its neutralizing activity against SARS‐CoV‐2 Omicron BA.2 variant. *n* = 6 biological replicates. E) Quantification of relative virus RNA copy number to assess aptamer 5’‐40 kDa PEG ‐5‐2‐15 with 2’‐F‐O modifications (PEG40K‐#5‐2‐15‐2’‐F‐O) for its neutralizing activity against SARS‐CoV‐2 Omicron BA.2 variant. *n* = 6 biological replicates. F) Quantification of relative virus RNA copy number to examine aptamer 5’‐Cholesterol‐40 kDa PEG‐#5‐2‐15 with 2’‐F‐O modifications (Chol‐PEG40K‐#5‐2‐15‐2’‐F‐O) for its neutralizing activity against SARS‐CoV‐2 Omicron BA.2 variant. *n* = 6 biological replicates. G) A schematic depicting the animal experiment to examine the prophylactic and therapeutic effect of intranasally (i.n.) delivered Chol‐PEG40K‐#5‐2‐15‐2’‐F‐O against live SARS‐CoV‐2 Omicron BA.2 variant in vivo. H,I) Quantification of virus titer of lung tissue by RT‐qPCR and FRNT to assess the prophylactic effect of aptamer Chol‐PEG40K‐#5‐2‐15‐2’‐F‐O against SARS‐CoV‐2 Omicron BA.2 variant. J,K) Quantification of virus titer of lung tissue by RT‐qPCR and FRNT to assess the therapeutic effect of aptamer Chol‐PEG40K‐#5‐2‐15‐2’‐F‐O against SARS‐CoV‐2 Omicron BA.2 variant. NC‐RNA apt: the negative control RNA aptamer. FRNT assay: focus reduction neutralization test. Readouts from negative controls served as 100% for normalization. IC50s were indicated. Data show mean ± SD. For in vivo experiment, *n* = 6 biological replicates. ^**^ *p* < 0.01, ^***^ *p* < 0.001, ^****^ *p* < 0.0001, two‐tailed *t*‐tests. GraphPad Prism 8.0.1 software was used for statistical analysis.

Next, we examined whether these two aptamers could neutralize infection from live SARS‐CoV‐2 virus in a Biological Safety Level‐3 (BSL‐3) laboratory, considering that their binding to RBD might interrupt the interaction of spike protein with the human Angiotensin‐Converting Enzyme II (hACE2) receptor (**Figure** **4**A). Consistent with binding activity to the pseudoviruses (Figure 3M), both aptamers showed dose‐dependent inhibition against SARS‐CoV‐2 Omicron BA.1 variant (Figure 4B). The aptamer #5‐2‐15 showed stronger potency, with an IC_50_ lower than 0.6 ng mL^−1^. Therefore, we also examined its neutralizing activity against live Delta variant. In fact, the aptamer #5‐2‐15 is also effective against Delta variant, although less efficient than that against Omicron BA.1 variant, with an IC_50_ of 20.28 ± 0.38 ng mL^−1^ (Figure 4C).

Native RNA is vulnerable to RNase mediated degradation. To this end, we replaced 2’ position of pyrimidines with fluorin (F) and purines with *O*‐methyl (O), respectively, to gain resistance.^[^ ^1^ ^]^ Interestingly, 2’‐F modified aptamers, but not 2’‐O, exhibited consistent higher binding signal to the recombinant RBD protein of Omicron BA.2, the variant prevailing while this manuscript is being prepared (Figure S5A,B, Supporting Information). And two types of modifications in aptamer #2‐1‐18 and #5‐2‐15, when combined, showed synergy in enhancing binding (Figure S5A,B, Supporting Information). Further, we conducted Electrophoresis gel‐Mobility Shift Assay (EMSA) to confirm their binding activity (Figure S5C, Supporting Information).^[^ ^50^ ^]^ When using recombinant RBD protein of the original SARS‐CoV‐2, 2’‐F‐O modifications of both aptamers led to enhanced shift, indicating stronger binding affinity (Figure S5D, Supporting Information). No shift was observed when using a negative control RNA aptamer, an indication of specificity (Figure S5D, Supporting Information). The binding specificity of 2’‐F‐O modified aptamers against both the original and Omicron BA.2 RBD were further validated by a RBD dose dependent shift and by dose dependent competition from unlabeled “cold” aptamers (Figure S5E,F, Supporting Information).

Considering the gradual dominance of Omicron BA.2 variant while this work is being conducted and the higher binding signal and neutralizing activity of the aptamer #5‐2‐15 among the leads in previous experiments, we next focused on the further development of #5‐2‐15 against live Omicron BA.2 variant. Data showed consistently potent neutralizing activity upon #5‐2‐15‐2’‐F‐O modifications, with an IC_50_ of 0.56 ± 0.64 ng mL^−1^ (Figure 4D). Polyethylene glycol with a 40 kDa molecular weight (PEG40K) is the most used to conjugate an aptamer therapeutic in the clinic,^[^ ^1^, ^51^, ^52^, ^53^, ^54^, ^55^, ^56^ ^]^ because of significant extension of aptamers’ half‐life from 24 to 96 h and great improvement of in vivo bioavailability.^[^ ^1^, ^57^, ^58^, ^59^ ^]^ Moreover, it was recently reported cholesterol conjugation provided most effective inhibition against SARS‐CoV‐2 for a peptide inhibitor of virus membrane fusion with host cells, possibly due to cholesterol (Chol) mediated cell surface presentation.^[^ ^60^ ^]^ We therefore linked the cholesterol‐PEG40K moiety to the 5’ end of #5‐2‐15‐2’‐F‐O (Chol‐PEG40K‐#5‐2‐15‐2’‐F‐O). PEG40K‐#5‐2‐15‐2’‐F‐O was also generated and examined. PEG40K and cholesterol conjugations, by itself or combined, again showed consistently potent neutralizing activity against live Omicron BA.2 variant (Figure 4E,F), with an IC_50_ of 3.10 ± 2.35 and 1.85 ± 0.82 ng mL^−1^, respectively. To validate whether activity is from the aptamer, we next examined these conjugating components without it, including cholesterol, PEG40K, and Chol‐PEG40K. Data showed that cholesterol alone could enhance SARS‐CoV‐2 infection, possibly because SARS‐CoV‐2 requires cholesterol for viral entry (Figure S6A, Supporting Information), an observation consistent with previous reports.^[^ ^61^, ^62^ ^]^ Neither PEG40K nor Cholesterol‐PEG40K showed an obvious effect on SARS‐CoV‐2 infection (Figure S6B,C, Supporting Information). Taken together, these data confirmed that, aptamer, not these chemical conjugations, is responsible for neutralizing activity against the BA.2 virus.

### 2.8 Prophylactic and Therapeutic Activity against Live Omicron BA.2 upon Intranasal Delivery

Having observed that PEG40K and cholesterol conjugation did not infer the aptamer's SARS‐CoV‐2 neutralizing action, we are encouraged to challenge these modalities for their in vivo activity, which, as far as we know, has not been reported so far.^[^ ^38^, ^39^ ^]^ We examined the prophylactic and therapeutic effects of Chol‐PEG40K‐#5‐2‐15‐2’‐F‐O aptamer against live Omicron BA.2 variant in vivo. Mice were treated with the Chol‐PEG40K‐#5‐2‐15‐2’‐F‐O modified‐aptamer intranasally 3 h before or after SARS‐CoV‐2 Omicron BA.2 variant virus infections to model prophylactic and therapeutic treatments, respectively. After 24 and 48 h, lung tissue was collected for virus titer quantification (Figure 4G). As shown in Figure 4H,I, viral titers after prophylactic administration are profoundly reduced when measured by either RT‐qPCR (Figure 4H) or in a functional Focus Reduction Neutralization Test (FRNT) (Figure 4I).^[^ ^63^ ^]^ The reduction is 94% on average at 24 h and persistently 91% on average at 48 h as determined by FRNT (Figure 4I). Viral titers upon therapeutic treatment are also profoundly reduced (Figure 4J,K) by 78% on average at 24 h and persistently reduced by 94% on average at 48 h by FRNT (Figure 4K). These results highlight the exciting potential for Chol‐PEG40K‐#5‐2‐15‐2’‐F‐O aptamer to be further developed into a prophylactic and therapeutic modality against the highly transmissible Omicron variant.

Interestingly, in contrast to similar effects in cell culture (Figure 4E), PEG40K‐#5‐2‐15‐2’‐F‐O aptamer without cholesterol conjugation loses activity against live Omicron BA.2 in vivo (Figure S7, Supporting Information), an indication of the requirement for efficient retention of the aptamer on the cell surface for its efficiency when delivered intranasally. This is consistent with a previous observation that cholesterol conjugation is required for activity of an intranasally delivered peptide inhibitor targeting SARS‐CoV‐2 membrane fusion with host cells.^[^ ^60^ ^]^

We also examined Chol‐PEG6‐#5‐2‐15‐2’‐F‐O, in which PEG40K was replaced with that of a lighter molecular weight, and of a faster turnover in vivo.^[^ ^59^ ^]^ As shown in Figure S8A (Supporting Information), Chol‐PEG6‐#5‐2‐15‐2’‐F‐O showed consistent Omicron BA.2 variant neutralizing activity in cell culture. Upon intranasal delivery to mice, its neutralizing activity was observed after 24 h but did not persist to 48 h (Figure S8B–E, Supporting Information), a phenomenon that could be explained by the shorter life‐span of PEG6 in vivo compared with that of PEG40K.^[^ ^59^ ^]^ Further, Chol and PEG40K modifications are also necessary for in vivo efficacy of a previously reported RNA aptamer PB6‐Ta (Figure S9, Supporting Information).^[^ ^33^ ^]^ Taken together, we identified 2’‐F‐O, Chol, and PEG40K modifications of the aptamer lead #5‐2‐15, which, when combined, rendered prophylactic and therapeutic activity against live Omicron BA.2 variant upon intranasal delivery in vivo.

### 2.9 Adaptation of CRISmers to Orthogonal CRISPR/Cas Systems, Selection Markers, and Host Species

**Figure 5:**
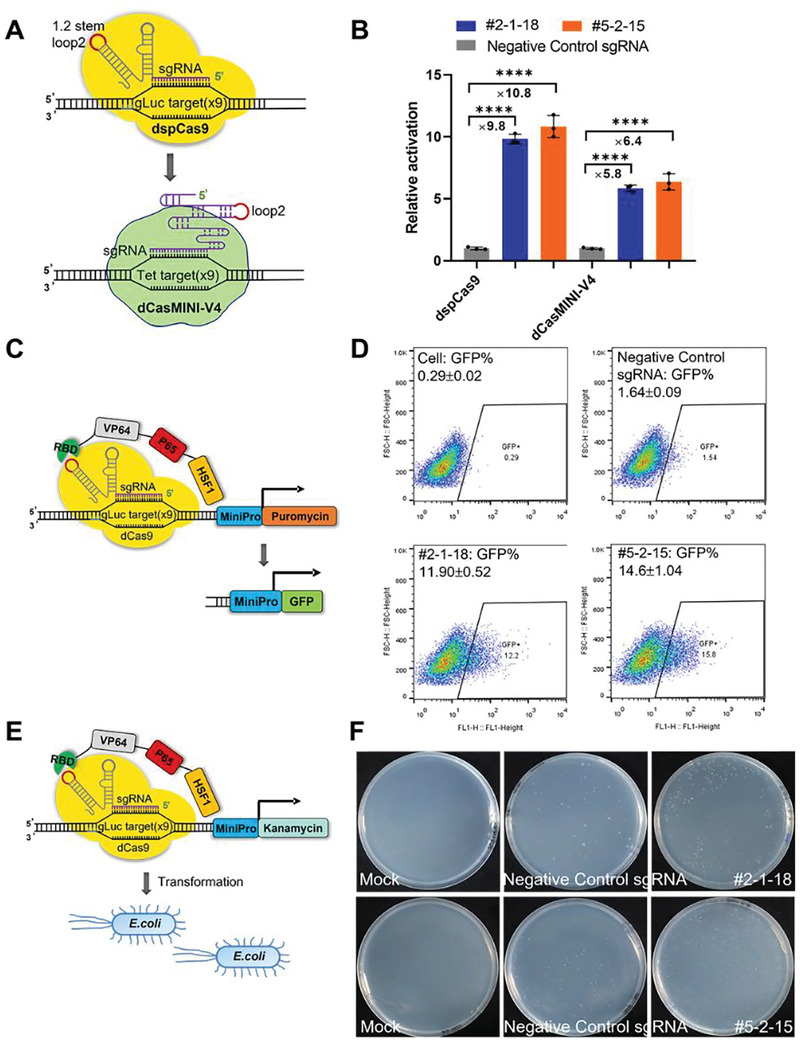
Adaptation of CRISmers to an orthogonal CRISPR/Cas, different selection markers and host species. A,B) dCasMINI‐V4, an evolved version of Un1Cas12f1 CRISPR/Cas system, was used in place of CRISPR/Cas9 in CRISmers. The aptamer sequence was inserted into the loop 2 of its sgRNA scaffold. The firefly‐luciferase reporter assay was used to examine activation. C,D) Switching the CRISmers selection marker to GFP. A negative control sgRNA used in luciferase reporter assay was gLuc target without any aptamer appendage (the sgRNA‐1.2 BsmBI scaffold, NC). was used as negative control. E,F) Switching host cells from human HEK293T to bacteria *E.coli*. Kanamycin was used for selection. Kanamycin in the LB agar plates was used at 100 µg mL^−1^ for #5‐2‐15 and 75 µg mL^−1^ for #2‐1‐18, respectively. Mock: plasmids control without kanamycin resistant gene. Exp: plasmids harboring #2‐1‐18 or #5‐2‐15 aptamer. Negative control sgRNA used in luciferase reporter assay was gLuc target without any aptamer appendage (the sgRNA‐1.2 BsmBI scaffold, NC). *n* = 3 biological replicates. ^****^ *p* < 0.0001, two‐tailed *t*‐tests. GraphPad Prism 8.0.1 software was used for statistical analysis.

Having successfully developed CRISmers and identified an aptamer lead effectively neutralizing SARS‐CoV‐2 Omicron BA.2 variant both in vitro and in vivo, we next asked whether the principle of CRISmers is broadly applicable by using two aptamer leads but switching to an orthogonal CRISPR/Cas system, selection marker, and host species. As examples for demonstration, we used Un1Cas12f1, a newly developed mini version CRISPR system with a much smaller size (**Figure** **5**A,B),^[^ ^64^, ^65^, ^66^ ^]^ GFP as a marker compatible with fluorescent sorting (Figure 5C,D; Figure S10, Supporting Information), kanamycin resistant gene as an alternative selection marker for antibiotic enrichment (Figure 5E,F), and *E. coli* as a bacterial host species (Figure 5E,F). Using aptamers #2‐1‐18 and #5‐2‐15, expected transactivation activity and enrichment of cells positive for selection markers were consistently observed (Figure 5), an indication of CRISmers’ broad compatibility and utility and their robustness and consistency in performance.

## 3 Conclusion

In summary, here we developed CRISmers, a novel system based on CRISPR/Cas for RNA aptamer screening that marries the two powerful RNA‐based systems. CRISmers adds a new tool to the CRISPR box, further unleashing its potential beyond the native nuclease function. Using CRISmers, we accomplished successful identification of aptamers targeting RBD of the SARS‐CoV‐2 spike protein. The aptamer leads are sensitive to detecting original SARS‐CoV‐2 pseudovirus as well as its Delta and Omicron variants. Strikingly, the leading aptamer #5‐2‐15 showed potent neutralizing activity against the live Omicron BA.2 variant virus, the dominant variant at the time of concluding this study, both in cell culture and in animal experiments. These results suggest its potential, when properly modified, for further developed as an RNA aptamer‐based prophylactic and therapeutic modality against the highly transmissible Omicron variant. Finally, using the two aptamer leads identified herein, we concluded this study by showcasing the potential broad compatibility and utility, consistent and robust performance of CRISmers by switching SpCas9 to new mini Un1Cas12f1, puromycin to GFP or kanamycin as selection markers, and host species from human HEK293T cells to bacterial *E. coli*.

CRISmers can be a powerful complement to the traditional SELEX, considering its unique characteristics. The entire screening process in our CRISmers system is carried out within cells, in which natural endogenous native biology conditions for folding and interaction of target proteins and RNAs would be better presented. The screening performance is also less likely to be affected by the experimental conditions such as buffer and temperature variations.^[^ ^1^ ^]^ And the background noise of CRISmers, which is embedded into the dead cells upon antibiotic selection, is easier to abolish from the system comparing with nucleic acids in solution.^[^ ^1^ ^]^ Meanwhile, what brings merit to CRISmers might also become limitations. Guide RNA appendage strategy limits its use only to RNA, not DNA aptamers. And due to native RNA production inside cells, nucleotide modification cannot be incorporated at the initial screening stage like SELEX does. Therefore, complementary use of CRISmers and SELEX might be necessary for many future studies.

While the current study used SpCas9 and Un1Cas12f1 as the CRISPR/Cas scaffolds, puromycin, kanamycin, and GFP as the selection markers, and human HEK293T cells and bacteria *E. coli* as hosts, similar strategies can be foreseen in other CRISPR species, selection markers, enrichment methods, host species, etc., thus further expanding its applicability and the target coverage. Screening can be further scaled up readily as industry‐scale mammalian cell culture or bacterial fermentation, used for recombinant protein, antibody production, etc., are routine nowadays.

In this study, we chose the RBD of SARS‐CoV‐2 as our very first target to test the performance of the CRISmers system, considering the urgency of the current global pandemic. To date, this is the first report that an aptamer targeting and neutralizing SARS‐CoV‐2 has ever been examined in vivo using live virus. Interestingly, we found that conjugations with both PEG with high molecular weight and cholesterol are necessary to maintain its SARS‐CoV‐2 neutralizing activity from cell culture to animal (Figure 4; Figures S7–S9, Supporting Information). Multiplexing nanoparticles might further enhance their potency in the future.^[^ ^37^, ^67^ ^]^ To our surprise, the aptamer lead #5‐2‐15 that we characterized most vigorously showed anti‐SARS‐CoV‐2 activity across all variants of concern from Delta to Omicron BA.2 that we have examined. Consistently, previous studies also observed other aptamers′ capability to avoid mutational escape.^[^ ^37^, ^67^ ^]^ This is very different from neutralizing antibodies, almost all of which are vulnerable to mutations.^[^ ^68^ ^]^ This highlights a possible inherent distinction between protein and RNA. RNA by nature might be more flexible in conformational adaptation which might make it less vulnerable to mutations. CRISPR systems might be good examples. Guide RNAs, naturally occurring aptamers, can be swapped between CRISPR species within the same class and can still work fine with Cas proteins from another CRISPR system.^[^ ^69^ ^]^

In theory, CRISmers might be better suited to target intracellular proteins as they are localized within the cellular nucleus in the system in order to activate a selection reporter gene. However, on the other hand, as the delivery technologies for exogenous RNA evolve, which is a key contributing factor to the success of mRNA vaccines, intracellular targets will become more accessible to RNA therapeutics.^[^ ^70^ ^]^ Last but not least, CRISmers may also find broad utility in studying the fundamental RNA‐protein interaction in general. Above all, we would anticipate a wide range of applications of CRISmers in biomedical research beyond the COVID‐19 era.

## 4 Experimental Section

### Reagents

His‐tagged RBD of SARS‐CoV‐2 Spike Protein (S1N‐C52H3), His‐tagged RBD of SARS‐CoV‐2 Omicron BA.2 Spike Protein (SPD‐C522g), Streptavidin‐HRP (STN‐NH115), and ELISA Buffer Set‐96T (EBS‐001) were purchased from ACROBiosystems (Beijing, China). High binding microplates (42 592) were purchased from Corning (Kennebunk, USA). DNA sequences were synthesized by GenScript (Nanjing, China). RNA sequences were synthesized by Sangon Biotech (Beijing, China). All media for cell culture were purchased from Life Technologies (Shanghai, China). All reagents for library construction were purchased from New England BioLabs (Ipswich, USA). Firefly luciferase assay kits (T003) were purchased from Vigorous Biotechnology (Beijing, China). Blasticidin, hygromycin, and puromycin were purchased from Invivogen (Toulouse, France). The Amplicon sequencing was performed in GENEWIZ (Suzhou, China). RNA extraction kits (0 654 358 8001) were purchased from Roche (Indianapolis, USA). RT‐qPCR kits (ZC‐HX‐201‐2) were purchased from BioGerm (Shanghai, China). Chemiluminescent EMSA kit was purchased from Beyotime (Shanghai, China). Native and modified aptamers were synthesized by GENERAL BIOL (Anhui, China) and Biosyntech (Suzhou, China). For chemical‐modified aptamers: 2’‐F: 2’ position of pyrimidines with fluorin modification; take aptamer #5‐2‐15 as an example: A**CUU**GG**CC**AA**UU**A**CC**G**U**AG**UUUUU**G**U**AG**UCU**G**C**AGGG**CC**AAG**U**G, 2’‐O: 2’ position of purines with 2’‐*O*‐methyl modification; take aptamer #5‐2‐15 as an example: **A**CUU**GG**CC**AA**UU**A**CC**G**U**AG**UUUUU**G**U**AG**UCU**G**C**AGGG**CC**AAG**U**G**, 2’‐F‐O: 2’ position of pyrimidines with fluorin and purines with 2’‐*O*‐methyl modifications.

### Plasmids

To generate 293T/dCas9 stable cell line, dCas9 was cloned from the pMSCV_LTR_dCas9_VP64_BFP plasmid (a gift from Stanley Qi & Jonathan Weissman, Addgene plasmid #46 912).^[^ ^71^ ^]^ Multiple activation domains, including VP64 (V), P65 (P), and HSF1 (H), were cloned from Addgene plasmids (VP64 was amplified from pLenti_EF1a_SOX2, a gift from Feng Zhang, Addgene plasmid #35 388;^[^ ^72^ ^]^ P65 was amplified from SP_dCas9_VPR, a gift from George Church, Addgene plasmid #63 798;^[^ ^73^ ^]^ HSF1 was amplified from plenti_MS2_P65_HSF1 _Hygro, a gift from Feng Zhang, Addgene plasmid 61 426).^[^ ^74^ ^]^ Human codon nucleotide sequences encoding the S glycoprotein, the subunits of the S glycoprotein (S1, amino acids 2 to 673, RBD amino acids 319 to 541, GISAID accession ID: EPI_ISL_414 631), Delta variant (GISAID accession ID: EPI_ISL_2 356 230), Omicron BA.1 variant (GISAID accession ID: EPI_ISL_6 825 398), Omicron BA.2 variant (GISAID accession ID: EPI_ISL_8 207 301), and the hACE2 were synthesized by GenScipt. The negative control RNA aptamer (NC‐RNA apt) used in ELONA, EMSA, and pseudovirus/live virus neutralization assays was provided by Sangon Biotech, whose sequence is ACUUGGCCAuuuuucuuuuucuuuuucuuuuuCUGCAGGGCCAAGUG. The negative control sgRNA (the sgRNA‐1.2 BsmBI scaffold, NC) used in the luciferase reporter assay was one without any aptamer appendage.

The 1 × gLuc/9 × gLuc target luciferase reporter plasmids (p1 × gLuc target‐miniPro‐firefly; p9 × gLuc target‐miniPro‐firefly) used in this study were constructed by inserting one copy or nine copies of the gLuc sgRNA target sequence upstream and firefly luciferase downstream of the mini promoter. The 9 × gLuc target puromycin reporter plasmid (p9 × gLuc target‐miniPro‐puromycin) was constructed by replacing firefly luciferase with puromycin. The aptamer appending gLuc sgRNA plasmids (phU6‐gLuc sgRNA‐1.1‐BsmBI; phU6‐gLuc sgRNA‐1.2 BsmBI) were constructed by inserting the gLuc sgRNA sequence in pre‐synthesized sgRNA 1.1 or sgRNA 1.2 backbone containing BsmBI sites in the tetra loop or stemloop 2, followed by incorporation of aptamers via BsmBI digestion and ligation. A GFP aptamer of canonical sequence was used in this study.^[^ ^46^ ^]^ The lentiviral shuttle plasmid for library construction (phU6‐gLuc sgRNA‐1.2 BsmBI‐EF1a‐NLS‐EcoRI‐VPH) was generated by cloning EF1a promoter‐nuclear localization signal (NLS)‐EcoRI‐VPH downstream of hU6‐gLuc sgRNA‐1.2 BsmBI. The 9 × gLuc target GFP reporter plasmid (p9 × gLuc target‐miniPro‐GFP) was constructed by puromycin with GFP. The 9 × gLuc target kanamycin reporter plasmid (p9 × gLuc target‐miniPro‐Kana^+^) was constructed by replacing puromycin resistant gene with kanamycin resistant gene, and then inserted all CRISmers system's components (phU6‐gLuc sgRNA‐1.2 aptamer‐EF1a‐NLS‐RBD‐VPH, 3 × NLS‐dpCas9, and the 9 × gLuc target‐miniPro‐kanamycin) into a plasmid (phU6‐gLuc sgRNA‐1.2 aptamer‐EF1a‐NLS‐RBD‐VPH‐P2A‐3 × NLS dspCas9‐3 × polyA‐9 × gLuc target‐miniPro‐kanamycin‐Amp promoter‐ampicillin) to verify the expression of kanamycin. dUn1Cas12f1 (dCasMINI‐V4) was cloned from pHR‐PGK‐SV40_NLS‐dCasMINI‐V4‐VPR‐c‐Myc_NLS‐mCherry‐WPRE plasmid (a gift from Stanley Qi, Addgene plasmid #176 269).^[^ ^64^ ^]^ The aptamer sequence was incorporated in the loop 2 of its sgRNA scaffold.^[^ ^64^ ^]^

### Cell Culture

HEK293T cells (ATCC) were maintained in Dulbecco's modified Eagle's Medium (DMEM) supplemented with 10% (v/v) fetal bovine serum (FBS), 2 mm GlutaMAX (Thermo Fisher), and 100 U mL^−1^ penicillin/streptomycin at 37 °C with 5%CO_2_. HEK293T/dCas9 stable monoclonal cell line was generated via infection of HEK293T with lentiviral particles containing the dCas9 expression cassette, followed by monoclonal picking, expansion, and examination. The HEK293T/dCas9/9 × gLuc puromycin reporter stable monoclonal cell line was produced via another round of infection to the monoclonal HEK293T/dCas9 cell line using lentiviral particles harboring the 9 × gLuc target puromycin reporter. Monoclones were picked, expanded, and examined as described in Figure S2A (Supporting Information). The polyclonal HEK293T/hACE2 stable cell line was generated by lentiviral transduction of HEK293T cells with pLenti‐CMV‐hACE2. Transient transfections were conducted using PEI (polysciences, Warrington) according to the manufacturer's recommended protocol.

### CRISmers Screening

*Lentiviral‐CRISmers Random Library Construction*: Human codon optimized nucleotide sequences encoding the RBD of Spike protein (S1, RBD amino acids 319 to 541, GISAID accession ID: EPI_ISL_414 631) were synthesized by GenScipt. The RBD was first inserted into the library shuttle vector phU6‐gLuc sgRNA‐scaffold‐1.2 BsmBI‐ scaffold‐polyT‐EF1a‐NLS‐linker‐EcoRI‐linker‐VPH at the EcoRI restriction sites. Then N20 pooled oligos flanked by two 40 bp overlapping sequences (AGCAAGTTAAAATAAGGCTAGTCCGTTATCAACTTGGCCAnnnnnnnnnnnnnnnnnnnnCTGCAGGGCCAAGTGGCACCGAGTCGGTGCTTTTTATCGA, synthesized by Sangon Biotech) were PCR amplified and cloned into the BsmBI sites of the library vector phU6‐gLuc sgRNA‐scaffold‐1.2 BsmBI‐scaffold‐polyT‐EF1a‐NLS‐linker‐EcoRI‐WT RBD‐EcoRI‐linker‐VPH with Gibson assembly ligation.^[^ ^75^, ^76^ ^]^ The number of PCR cycles was limited to 15 cycles to minimize PCR bias and byproducts. The high‐fidelity polymerase Phusion Hot Start Flex 2 × Master Mix (NEB, Ipswich, USA, M0536S) was used for PCR. Then the PCR reaction was run on a 2% agarose gel, and product ≈140 bp was purified using QIAquick PCR Purification (QIAGEN, Hilden, Germany, 28 104). After the gibson assembly, isopropanol precipitation was performed to purify and concentrate the library before pooled transformation. After 32 °C incubation for 18 h post‐transformation, all the surviving colonies were harvested,the pooled library was purified via Maxiprep according to manufacturer's instructions. Based on the calculation of the number of colonies, the diversity of the sgRNA‐aptamer library could be estimated to be 10^8^. Cloning of sub‐libraries in the following rounds followed the same protocol, while using amplicons from the genomic DNA of surviving cells after selection.

*CRISmers Random Library Lentivirus Production*: HEK293T cells were seeded and cultured to achieve optimal confluency (≈80–90%) for transfection. Lentiviral transgene expression plasmids, psPAX2 (Addgene plasmid #12 260), and pMD2.G (Addgene plasmid #12 259) were co‐transfected. After 6 h post‐transfection, culture plate medium was replaced with fresh DMEM containing 10% FBS, and cells were further cultured at 37 °C. Virus supernatant was harvested for 48 and 72 h post‐transfection, filtered with 0.45 µm syringe filters (PALL), and stored at −80 °C when necessary.

*CRISmers Random Library Lentivirus Transduction*: Lentiviral particles were used to infect the No.4‐53 monoclonal dCas/9 × gLuc‐Puro cell line. Suspended cell infection was conducted with 8 µg mL^−1^ polybrene (Sigma) added to the culture. The medium was changed after 8 h of infection. During the initial round of CRISmers, based on the calculation, 20 cells are presented for infection per sequence from the pooled library. And the pooled lentivirus particles were transduced at a Multiplicity of Infection (MOI of ≈3 to maximize the representation of the aptamer population in the infected cells. After the first round, most background RNA sequence is removed from the system, a coverage of >500 cells per sequence is maintained during sub‐library transductions and a lower MOI of ≈0.1‐0.2 is used to increase the probability that most cells receive only one aptamer.

*Puromycin Selection*: The cells were initially plated at low density and left for 48 h. After this, 2 µg mL^−1^ puromycin was added to select for puromycin‐resistant cells. The medium was renewed on alternate days until there were no viable cells in the control plate of non‐infected cells.

*Genomic DNA Extraction*: The puromycin‐resistant cells were subsequently cultured for an additional 5–7 days. The surviving cells were collected. Genomic DNA was extracted to amplify the aptamer sequences.

*Deep Sequencing and Analysis (Information read‐out)*: The N20 oligos region, flanked by two 40 bp overlapping sequences, was PCR amplified and deep sequenced by Genewiz. Sequences that were not at the correct size (140 bp; 20 bp 5′ primer +40 bp 5′ flanked region +20 bp random region +40 bp 3′ flanked region +20 bp 3′ primer) or did not contain the correct primer/flanked region sequences on each end were filtered out, resulting in over >4 × 10^6^ filtered sequences per round. These filtered sequences were then sorted by frequency.

To identify high affinity sequences, the top 0.1% high frequency sequences and the top 15 sequences with the highest fold‐of‐enrichment ratio of copy numbers in comparison were calculated and ranked against the previous round of screening,^[^ ^47^ ^]^ and they were tested in the aforementioned luciferase reporter assay as a secondary validation. The next cycle, when necessary, was initiated by subcloning the amplified aptamer sequences from the previous round into the original library shuttle construct.

### Validation of Top Hits (Luciferase Reporter Assay)

These identified aptamers were validated by the luciferase reporter assay as a secondary screen. Aptamers were synthesized by Sangon Biotech and cloned into the BsmBI sites of the vector phU6‐gLuc sgRNA‐scaffold‐1.2 BsmBI‐ scaffold‐polyT‐EF1a‐NLS‐linker‐EcoRI‐WT RBD‐EcoRI‐linker‐VPH. HEK293T/dCas9 cells were seeded at 2.5 × 10^4^ cells per well into 96‐well assay plates. And transient transfection was performed the next day using PEI with plasmids expressing 9 × gLuc target firefly/gaussia luciferase, hU6 ‐gLuc sgRNA‐scaffold‐1.2 aptamer‐scaffold‐polyT‐EF1a‐NLS‐linker‐EcoRI‐WT RBD‐EcoRI‐linker‐VPH, and a renilla luciferase expression plasmid as transfection control. The phU6‐gLuc sgRNA‐scaffold‐1.2 BsmBI‐ scaffold‐polyT‐EF1a‐NLS‐linker‐EcoRI‐WT RBD‐EcoRI‐linker‐VPH plasmid was used as a negative control. A total of 200 ng of DNA was transfected, and each plasmid was transfected in equal amounts. The medium was replaced with fresh medium 6 h after transfection. The firefly/gaussia and renilla luciferase activities were recorded sequentially by Luminescence Counter (Perkin Elmer) 48 h later.

### Puromycin Reporter Assay

The hU6‐gLuc sgRNA‐1.2 GFP apt‐EF1a‐NLS‐GFP‐VPH lentivirus (PC) and the hU6‐gLuc sgRNA‐1.2 BsmBI scaffold ‐EF1a‐NLS‐GFP‐VPH (NC) were packaged into lentivirus particles as described above. Each HEK293T/dCas9/9 × gLuc puromycin reporter monoclonal was transduced with these particles and selected with escalating concentrations of puromycin (low: 0.5 µg mL^−1^, medium: 1 µg mL^−1^, high: 2 µg mL^−1^). The number of surviving clones was counted.

### GFP Reporter Assay

HEK293T cells were seeded at 15.625 × 10^4^ cells per well into 24‐well assay plates. And transient transfection was performed the next day using PEI with plasmids expressing 9 × gLuc target GFP, hU6‐gLuc sgRNA‐1.2 #2‐1‐18 aptamer, or hU6‐gLuc sgRNA‐1.2 #5‐2‐15 aptamer, and dCas9. A total of 800 ng of DNA was transfected, and each plasmid was transfected in equal amounts. The medium was replaced with fresh medium 6 h after transfection. GFP^+^ cells were counted using a flow cytometry Calibur cell analyzer. Cells were first gated by FSC (Forward Scatter) versus SSC (Side scatter) to exclude cell debris. The FITC (FL1) channel was selected without compensation.

Cells were then gated on the FITC (FL1) channel, where the threshold was set by non‐transfected control cells. Gates are drawn to define positive cells on the basis of negative cell controls (Figure S10, Supporting Information).

### Examination of CRISmers in *E. Coli*

The Mock group plasmid (unrelated plasmids control without kanamycin resistant gene), NC group plasmid (kanamycin reporter plasmid but with a negative control sgRNA (NC) used in luciferase reporter assay was gLuc target without any aptamer appendage (the sgRNA‐1.2 BsmBI scaffold), and experiment group (kanamycin reporter plasmid with aptamer) were transformed, and all LB agar plates of ampicillin were incubated overnight at 32 °C for 14–16 h, then transferred to 1 mL centrifuge tube with 600 µL LB medium with 100 µg mL^−1^ ampicillin and shaken at 32 °C for 3–4 h with 200 rpm. Then 100 µL culture was plated onto a LB agar plate with 100 µg mL^−1^ ampicillin and 100 µg mL^−1^ kanamycin (#5‐2‐15) or 75 µg mL^−1^ kanamycin (#2‐1‐18). These LB agar plates were cultured at 32 °C for another 14–18 h.

### SARS‐CoV‐2 Pseudovirus Production

HEK293T cells were seeded and cultured to achieve optimal confluency (≈80–90%) for transfection and were co‐transfected with 2 µg of plasmid encoding CMV‐luciferase, 0.4 µg of Spike or Delta or Omicron, 0.2 µg of TAT, 0.2 µg of Rev and 0.2 µg of GAGpol in a 6‐well plate each using PEI. After 6 h post‐transfection, the culture plate medium was replaced with fresh DMEM containing 10%FBS. Virus supernatant was harvested for 48 and 72 h post‐transfection, filtered with 0.45‐µm syringe filters (PALL), and stored at −80 °C when necessary.

### ELONA Assay

For the protein‐based ELONA assay, 96‐well high‐binding polystyrene microplates were pre‐coated with 250 ng RBD proteins at 4 °C overnight. Then the plates were washed with wash buffer (PBS with 0.05% (v/v) Tween‐20, pH 7.4) three times and blocked with blocking buffer (wash buffer with 2% (w/v) bovine serum albumin) for 30 min at 37 °C. Serial dilutions of biotinylated RNA aptamers in sterilized dH_2_O were added, and incubation lasted for 1 h at 25 °C. The plates were then washed with wash buffer three times. Streptavidin‐HRP ( 0.1 µg mL^−1^) was added and incubated for 30 min at room temperature. Substrate buffer was added after washing with wash buffer three times and incubated for 20 min at room temperature, followed by addition of stop buffer (1 m sulfuric acid). The absorbance of optical density at 450 nm (OD450) was measured using a microplate reader. The *Kd* values of aptamers were estimated using the following equation.

(1) $$Y=B_{\max}*X/\left(\mathrm{Kd}+X\right)$$

The *Y* represents the mean value of OD450, Bmax is the maximal value of OD450, and X is the concentration of the biotinylated aptamer. GraphPad Prism 8.0.1 software was used for statistical analysis.

As for ELONA using pseudovirus, different titers of the Spike pseudovirus and its variants Delta and Omicron were pre‐immobilized on microplates and then treated with 100 nM biotinylated aptamers. The process that follows is the same with the protein‐based ELONA. Pseudovirus titration was conducted following a previous report.^[^ ^77^ ^]^

### EMSA Assay

Electrophoretic Mobility Shift Assays (EMSAs) were performed according to the protocol provided by the Chemiluminescent EMSA kit (Beyotime Biotechnology, China). The fixed concentration of biotinylated aptamers (1 pmol) with varying ratios of SARS‐CoV‐2 WT RBD or SARS‐CoV‐2 Omicron RBD protein (1:0, 1:1, 1:10, 1:20, 1:50) in EMSA binding buffer were incubated at room temperature for 30 min. Besides, a fixed concentration of aptamers (1 pmol) and a 1:25 ratio of SARS‐CoV‐2 WT RBD or a 1:50 ratio of SARS‐CoV‐2 Omicron RBD protein with varying cold aptamers (non‐biotinylated aptamers, 1:1, 1:2, 1:100, 1:200) in EMSA binding buffer was incubated at room temperature for 30 min. After incubation, the samples were mixed with EMSA loading buffer, and the samples were run on a 4% polyacrylamide gel (4 mL 40% acrylamide/bis‐acrylamide, 1 mL 1 × TBE, 150 µL 10% APS, 10 µL TEMED, 625 µL 80% glycerol, and dH2O up to 20 mL) at 100 V for 1 h and then transferred to a N^+^ nylon membrane. After electrophoretic transfer, crosslinking was carried out with a hand‐held UV lamp for 5–10 min. Then these biotin‐labeled aptamers were detected by chemiluminescence.

### SARS‐CoV‐2 Live Virus Neutralization Assay

The experiment was performed in the Biological Lafety Level‐3 laboratory (BSL‐3) at the Shenzhen Center for Disease Control and Prevention. All experiments involving live SARS‐CoV‐2 virus followed the approved standard operating procedures of the Shenzhen Center for Disease Control and Prevention BSL‐3. The SARS‐CoV‐2 Delta virus, and Omicron BA.1 and BA.2 viruses were isolated from nasopharyngeal aspirate specimens of patients by Shenzhen Center for Disease Control and Prevention (SARS‐CoV‐2/shenzhen/09/2022 (Delta); SARS‐CoV‐2/shenzhen/08/2022 (Omicron BA.1); SARS‐CoV‐2/shenzhen/13/2022 (Omicron BA.2)).

The VERO cells were seeded into 96 microplates (1.5 × 10^4^ cells per well) and cultured overnight. The aptamers were incubated with SARS‐CoV‐2 virus in 2% FBS/DMEM at a final volume of 100 µL per well at indicated concentrations in a 5% (v/v) CO_2_, 37 °C incubator for 1 h. Medium containing aptamers and live viruses was then transferred to the VERO cell culture plates. After 2 h of incubation, the medium was replaced with 2% FBS/DMEM fresh medium, and culture was continued. After 48 h, media were harvested. RNA was extracted, and quantitative reverse‐transcription PCR (RT‐qPCR) was performed to quantitate the viral RNA. The relative virus RNA copy number was analyzed by nonlinear regression to calculate the IC_50_ value using GraphPad Prism 8.0.1 software.

### Ethics Statement

All protocols of animal experiments were approved by the Institutional Animal Care and Use Committees of Guangzhou Medical University.

### Animal Experiment

All work with the SARS‐CoV‐2 Omicron BA.2 variant virus was conducted in the Guangzhou Customs District Technology Center Biosafety Level 3 (BSL‐3) Laboratory. The SARS‐CoV‐2 Omicron variant, BA.2, was used to infect wild type BALB/c mice.^[^ ^78^ ^]^ Specific pathogen‐free 5–6 week old female BALB/c mice were lightly anesthetized with isoflurane and intranasally challenged with 1 × 10^5^ FFU of BA.2 live virus. For the prophylactic group, mice were intranasally administered with modified RNA aptamers (10 µg each mouse) dissolved in dH_2_O (75 µL each nostril) by a dropper 3 h before infection. For therapeutic group, mice were intranasally treated with modified RNA aptamers at 3 h post infection. After 24 and 48 h, mice were subjected to anesthesia and sacrificed before harvesting lung tissue for virus titering. Lungs were removed into PBS and homogenized. Clarified supernatants were harvested. RNA was extracted, and RT‐qPCR was performed to quantitate the viral RNA. Also, virus titers in clarified supernatants were assayed in Vero E6 cells and presented as FFU per gram of tissue using the FRNT assay.^[^ ^63^ ^]^ *n* = 6 biological replicates.

### Statistical Analysis

Data show mean ± SD. *n* = 3 biological replicates unless otherwise noted. *n* = 6 biological replicates in some of in vitro and all in vivo live virus neutralization experiments (Figure 4D,E,F,H,I,J,K; Figures S7–S9, Supporting Information). ^*^ *p* < 0.05, ^**^ *p* < 0.01, ^***^ *p* < 0.001, ^****^ *p* < 0.0001, two‐tailed *t*‐tests. n.s., no significant difference. GraphPad Prism 8.0.1 software was used for statistical analysis.

## Conflict of Interest

A provisional patent covering the CRISmers system and aptamers identified herein has been filed.

## Author Contributions

J.Z. and A.Z contributed equally to this work. Y.W. conceived and supervised the study. J.Z., R.Z., B.Z., X.T., and Y.W. designed experiments. J.Z., M.M., J.Q., A.Z., S.F., Y.S., and M.X. performed experiments and acquired the data. J.Z., J.Z., J.Z., R.Z., B.Z., X.T., and Y.W. analyzed data. J.Z., X.T., J.Z., B.Z., and Y.W. wrote and revised the manuscript.

## Supporting information

Supporting Information

## Data Availability

The data that support the findings of this study are available from the corresponding author upon reasonable request.
