# Supplementary material for: Repurposing CRISPR/Cas to Discover SARS‐CoV‐2 Detecting and Neutralizing Aptamers
Source: Adv Sci (Weinh). 2023 May 19;10(22):2300656. doi: 10.1002/advs.202300656 (PMC10401102; doi:10.1002/advs.202300656)
Supplement: Supplementary file 1 — Supporting Information [file ADVS-10-2300656-s001.pdf]

ADVANCED SCIENCE
Open Access

Supporting Information

for *Adv. Sci.*, DOI 10.1002/advs.202300656

Repurposing CRISPR/Cas to Discover SARS-CoV-2 Detecting and Neutralizing Aptamers

Ju Zhang, Airu Zhu, Miao Mei, Jing Qu, Yalan Huang, Yongshi Shi, Meiying Xue, Jingfang Zhang, Renli Zhang, Bing Zhou*, Xu Tan*, Jincun Zhao* and Yu Wang**

Supporting Information

Repurposing CRISPR/Cas to Discover SARS-CoV-2 Detecting and Neutralizing Aptamers

Ju Zhang, Airu Zhu, Miao Mei, Jing Qu, Yalan Huang, Yongshi Shi, Meiying Xue, Jingfang Zhang, Renli Zhang, Bing Zhou*, Xu Tan*, Jincun Zhao*, Yu Wang**

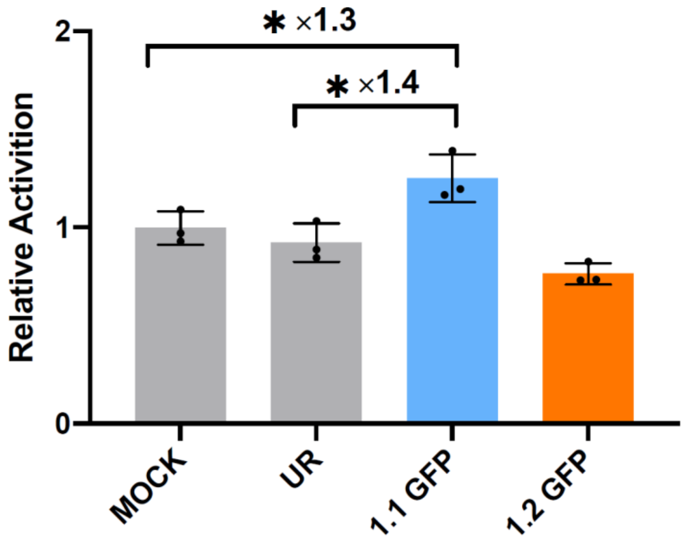

Figure S1. Key parameter characterization of a CRISPR/Cas9 based aptamer screening system. Results of a luciferase assay shown in Figure 1E, but using 1 repeat of gLuc sgRNA target sequence (1× gLuc). Mock: transient transfection of control plasmids. UR: gLuc sgRNA 1.2-the negative control sgRNA used in luciferase reporter assay was gLuc target without any aptamer appendage (the sgRNA-1.2 BsmBI scaffold). N=3 biological replicates. *$P<0.05$, two-tailed *t*-tests. GraphPad Prism 8.0.1 software were used for statistical analysis.

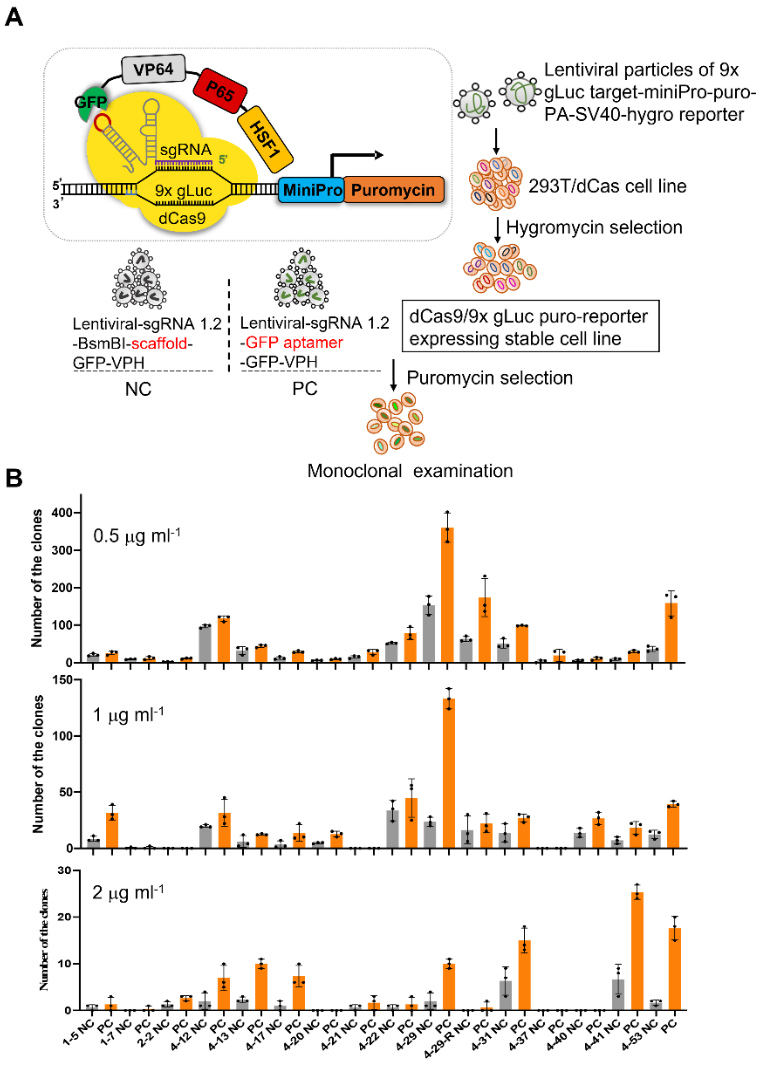

Figure S2. Construction and screening of monoclonal dCas9/9× gLuc-Puro cell lines. A) Schematic illustration of the gLuc puromycin reporter assay using GFP aptamer and the process of screening dCas9/9× gLuc-puro monoclonal cell lines. B) Results from a screen of dCas9/9× gLuc-puro monoclonal cell lines by quantifying the number of surviving clones after puromycin selection. Puromycin was applied at three different concentrations as shown in the graphs. NC: negative control; PC: positive control. n=3 biological replicates.

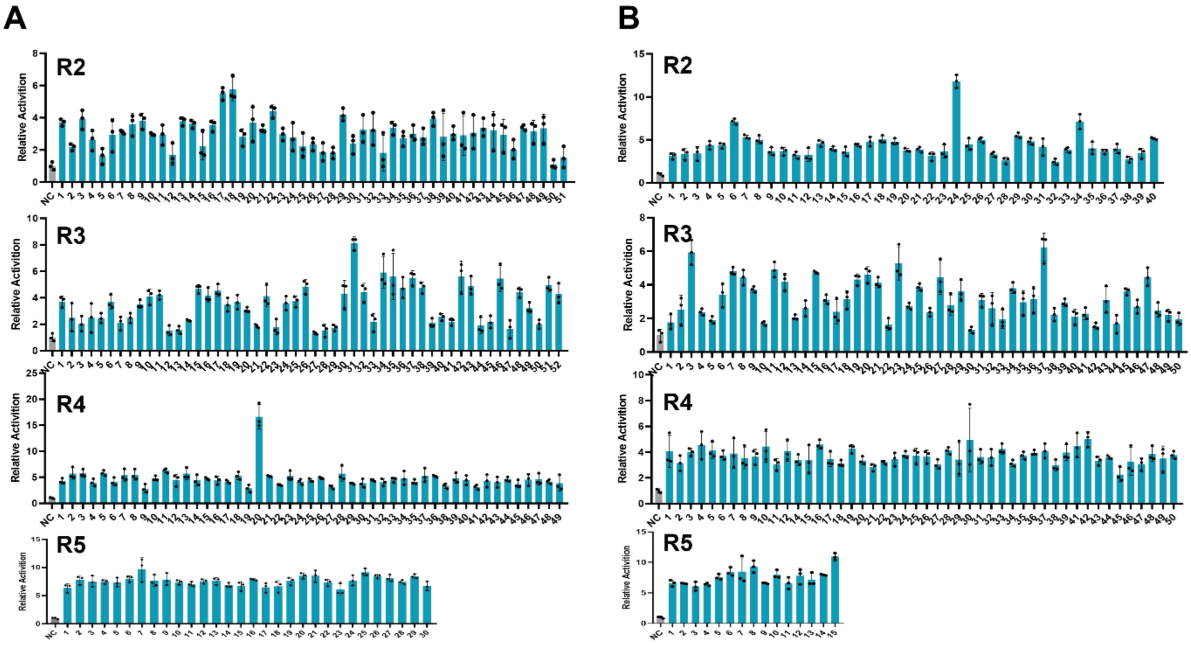

Figure S3. Secondary screens using luciferase reporter assay. The top 0.1% frequency sequences and the high enrichment-fold sequences derived from deep sequencing of aptamer amplicons harvested from round 2 (R2), R3, R4, and R5 were screened. Two parallel CRISmers primary screens were conducted (presented separately in A and B). NC: gLuc sgRNA 1.2-the negative control sgRNA used in the luciferase reporter assay was gLuc target without any aptamer appendage (the sgRNA-1.2 BsmBI scaffold). n=3 biological replicates.

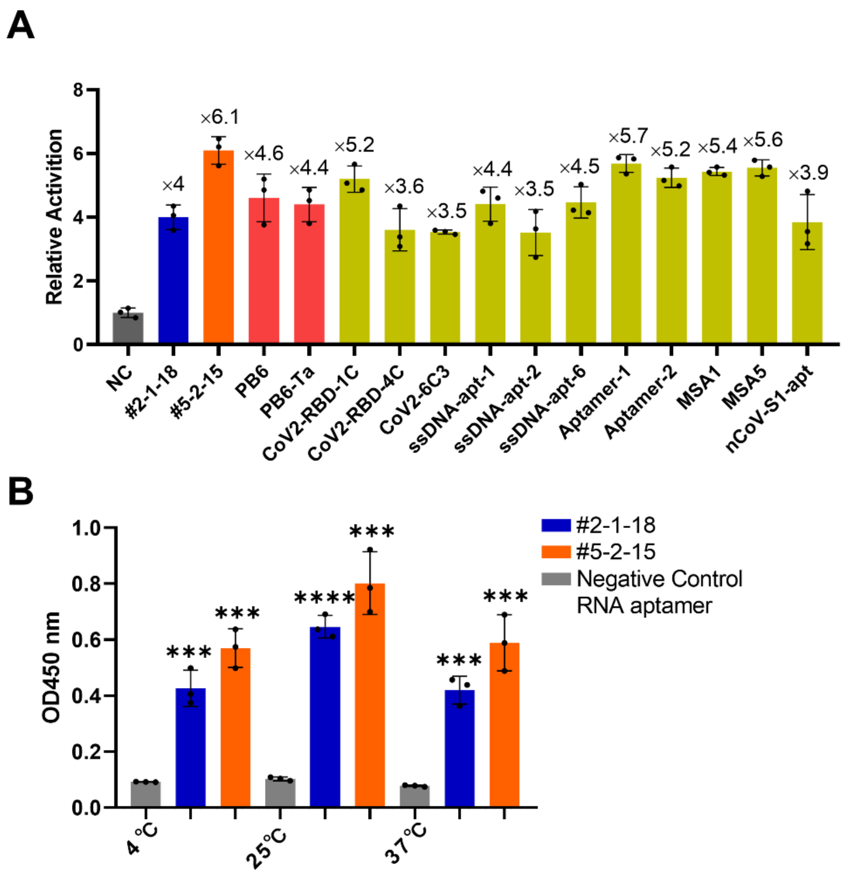

Figure S4. Examination of aptamer hits. A) Results of a luciferase reporter assay (Figure 1D) to examine aptamer hits and previously reported RNA (in red) and DNA aptamers (in dark yellow) identified from SELEX.[24-26, 28, 30, 33] NC: a negative control sgRNA used in the luciferase reporter assay was gLuc target without any aptamer appendage (the sgRNA-1.2 BsmBI scaffold). B) Examination of binding activity of aptamer leads with RBD at 4℃, 25℃, and 37℃ by ELONA. A negative control RNA aptamer was used as a negative control. N=3 biological replicates. ***P<0.001, ****P<0.0001, two-tailed t-tests. GraphPad Prism 8.0.1 software were used for statistical analysis.

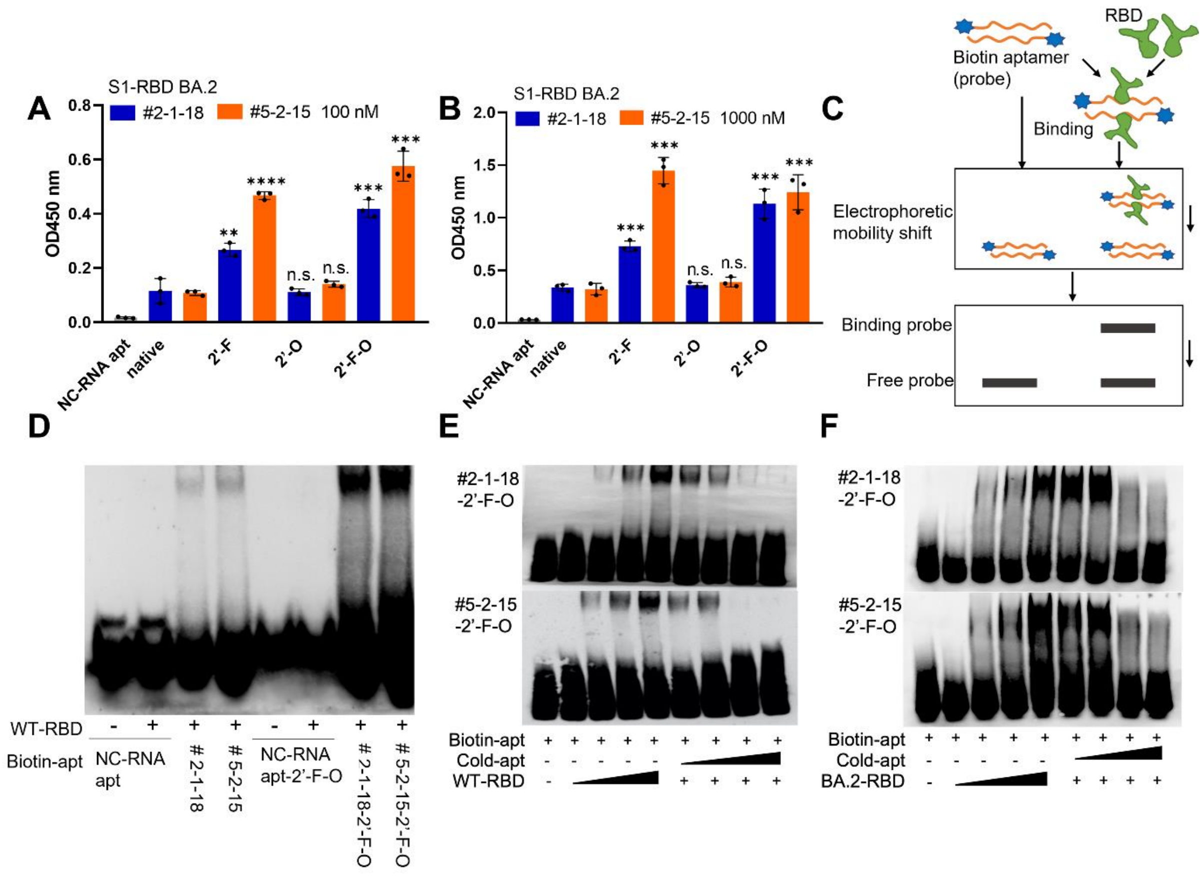

Figure S5. Examination of binding activity and specificity of modified aptamer leads with RBD. A, B) Examinations of native and modified aptamers of their binding activity to recombinant Omicron BA.2 RBD of SARS-CoV-2 Spike protein (250 ng) *via* ELONA, NC-RNA apt: the negative control RNA aptamer, native: native RNA aptamers with no modification, 2'-F: 2' position of pyrimidines with fluorin modification, 2'-O: 2' position of purines with 2'-*O*-methyl modification, 2'-F-O: 2' position of pyrimidines with fluorin and purines with 2'-*O*-methyl modifications. Aptamers were used with concentrations of 100 nM (A) and 1000 nM (B) respectively. C) A schematic depicting the Electrophoresis gel-Mobility Shift Assays (EMSA). D) EMSA results to compare native aptamers and 2'-F-O modified aptamers against recombinant RBD of the original SARS-CoV-2 Spike protein. E, F) EMSA examination of RBD dose dependent binding activity and specificity of modified aptamers against recombinant RBD of the original SARS-CoV-2 Spike protein (E) and Omicron BA.2 (F). A negative control RNA aptamer was used as a negative control. NC-RNA apt: the negative control RNA aptamer, NC-RNA apt-2'-F-O: the negative control RNA aptamer with 2'-F-O modification, Biotin-apt: biotinylated aptamer, Cold-apt: aptamer without biotin modification. N=3 biological replicates. $^{**}P<0.01$, $^{***}P<0.001$, $^{****}P<0.0001$, two-tailed *t*-

tests. n.s., no significant difference. All are compared with the respective native aptamer. GraphPad Prism 8.0.1 software were used for statistical analysis.

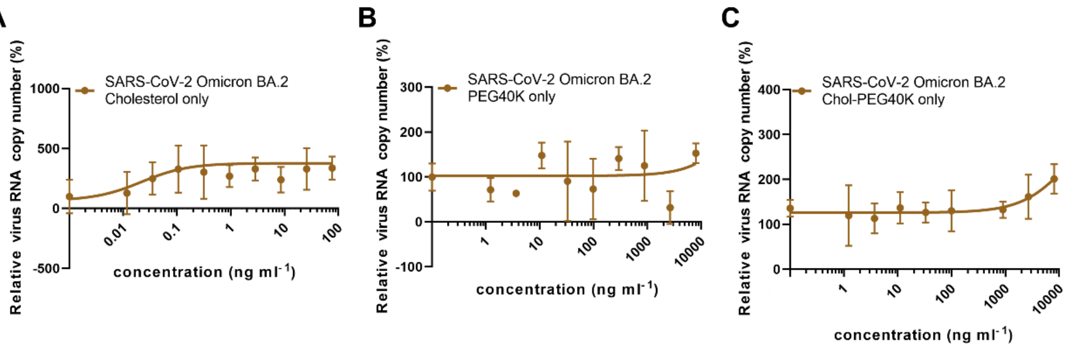

Figure S6. Examination of aptamer conjugating components for their activity upon live Omicron BA.2 variant. A) Quantification of relative virus RNA copy number of Cholesterol alone for its effect on SARS-CoV-2 Omicron BA.2 variant. B) Quantification of relative virus RNA copy number of 40kDa PEG alone for its activity on SARS-CoV-2 Omicron BA.2 variant. C) Quantification of relative virus RNA copy number of Cholesterol-40kDa PEG for its activity on SARS-CoV-2 Omicron BA.2 variant. n=3 biological replicates.

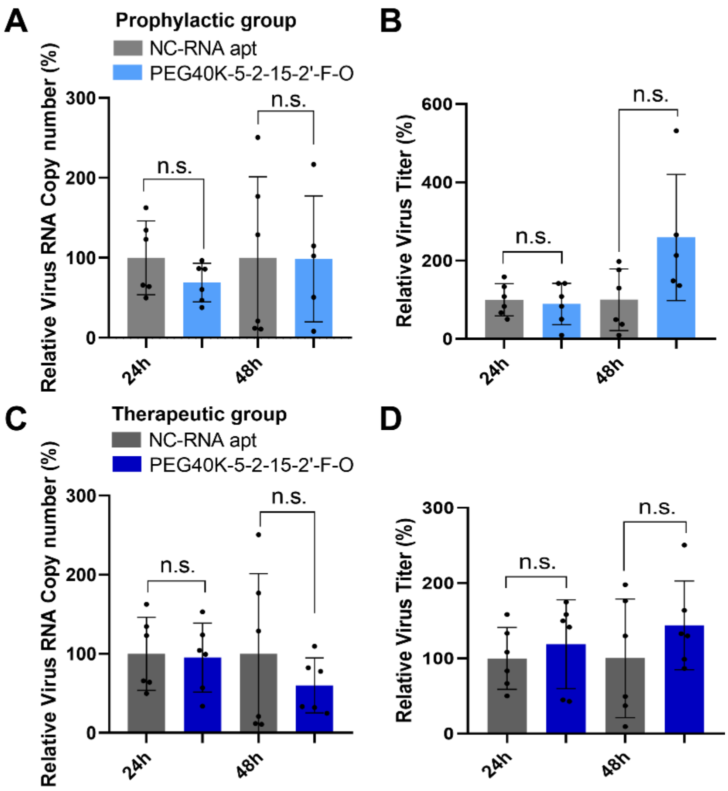

Figure S7. Examination the prophylactic and therapeutic activities of intranasally (i.n.) delivered PEG40K-#5-2-15-2'-F-O aptamer against live Omicron BA.2 variant *in vivo.* A, B) Quantification of virus titer of lung tissue by RT-qPCR (A) and FRNT (B) to assess the

prophylactic effect of aptamer PEG40K-#5-2-15-2'-F-O against SARS-CoV-2 Omicron BA.2 variant. C, D) Quantification of virus titer of lung tissue by RT-qPCR (C) and FRNT (D) to assess the therapeutic effect of aptamer PEG40K-#5-2-15-2'-F-O against SARS-CoV-2 Omicron BA.2 variant. FRNT assay: Focus Reduction Neutralization Test. Readouts from negative controls served as 100% for normalization. NC-RNA apt: the negative control RNA aptamer. N=6 biological replicates. n.s., no significant difference, two-tailed *t*-tests. GraphPad Prism 8.0.1 software were used for statistical analysis.

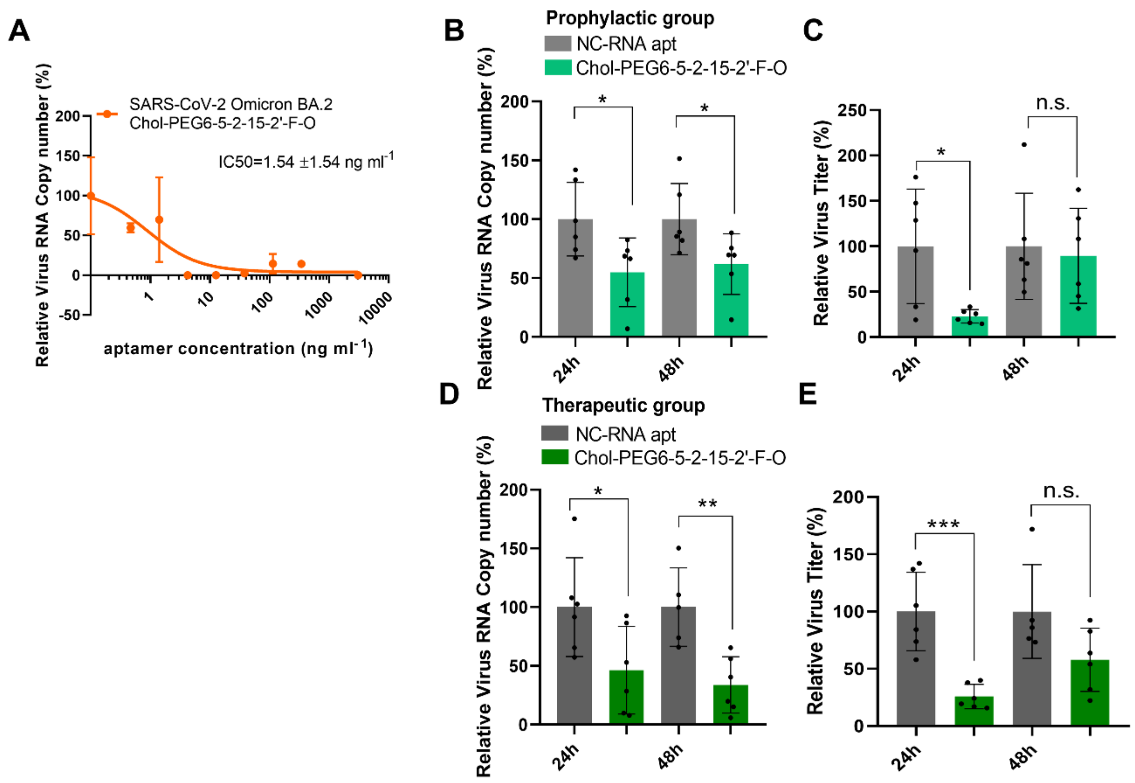

Figure S8. Examination of Chol-PEG6-#5-2-15-2'-F-O against live Omicron BA.2 variant in cell culture and animal experiments. A) Quantification of relative virus RNA copy number to examine Chol-PEG6-#5-2-15-2'-F-O for its neutralizing activity against live SARS-CoV-2 Omicron BA.2 variant in cell culture. n=3 biological replicates, Data show mean ± SD. B, C) Quantification of virus titer of lung tissue by RT-qPCR (B) and FRNT (C) to assess the prophylactic effect of Chol-PEG6-#5-2-15-2'-F-O against live SARS-CoV-2 Omicron BA.2 variant when delivered intranasally *in vivo*. D, E) Quantification of virus titer of lung tissue by RT-qPCR (D) and FRNT (E) to assess the therapeutic effect of Chol-PEG6-#5-2-15-2'-F-O against live SARS-CoV-2 Omicron BA.2 variant when delivered intranasally *in vivo*. FRNT assay: Focus Reduction Neutralization Test. Readouts from negative controls served as 100% for normalization. IC50s were indicated. NC-RNA apt: the negative control RNA aptamer, Data show mean ± SD. N=6 biological replicates. *P<0.05,

**P<0.01, ***P<0.001, n.s., no significant difference, two-tailed *t*-tests. GraphPad Prism 8.0.1 software were used for statistical analysis.

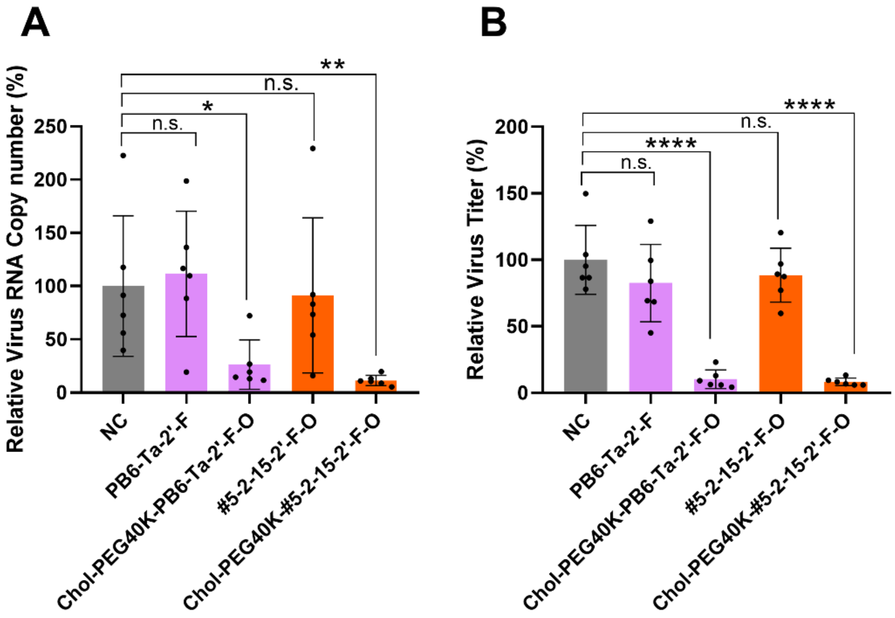

Figure S9. Examination the therapeutic effect of intranasally (i.n.) delivered PB6-Ta-2'-F, Chol-PEG40K-PB6-Ta-2'-F-O, #5-2-15-2'-F-O and Chol-PEG40K-#5-2-15-2'-F-O aptamers against live Omicron BA.2 variant *in vivo* (Figure 4G). A, B) Quantification of virus titer of lung tissue by RT-qPCR (A) and FRNT (B) to assess the therapeutic effect of aptamers against live SARS-CoV-2 Omicron BA.2 variant 48 hours post administration. FRNT assay: Focus Reduction Neutralization Test. Readouts from negative controls served as 100% for normalization. NC-RNA apt: the negative control RNA aptamer. N=6 biological replicates. *P<0.05, **P<0.01, ****P<0.0001, n.s., no significant difference, two-tailed *t*-tests. GraphPad Prism 8.0.1 software were used for statistical analysis.

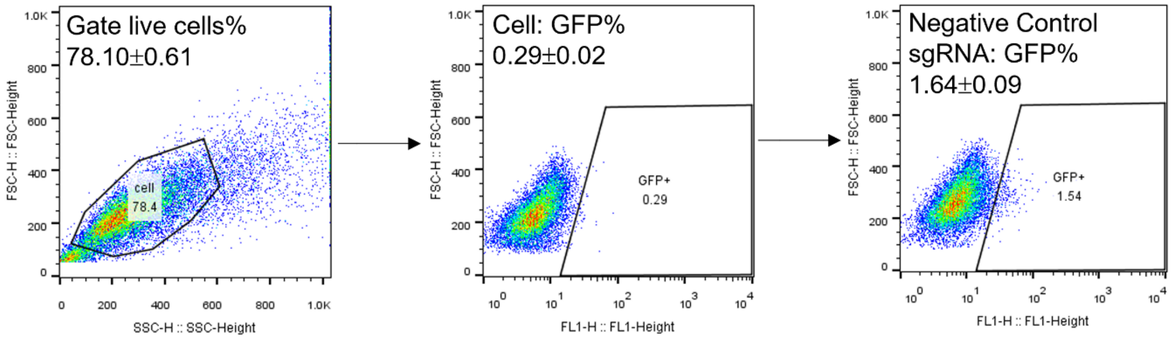

Figure S10. Cells gating of Flow cytometry experiment. Cells were gated of FSC (Forward Scatter) vs SSC (Side scatter) to exclude cell debris. The FITC (FL1) channel was selected without compensation. Cells were then gated on FITC (FL1) channel, where the threshold was

set by non-transfected control cells. Gates are drawn to define positive cells on the basis of negative cell controls. The negative control sgRNA group was an example to illustrate. N=3 biological replicates. GraphPad Prism 8.0.1 software were used for statistical analysis.

Table S1

Sequences of aptamers characterized in this study

Aptamer ID	Full length aptamer sequences (5'-3')
NC-RNA apt	ACUUGGCCAUUUUUCUUUUUCUUUUUCUUUUCUGCAGGGCCAAGUG
#2-1-17	ACUUGGCCACUCGGUAUCUAGUGCGCACUCUGCAGGGCCAAGUG
#2-1-18	ACUUGGCCACGUCGACUGAAAUCCUCUUCCUGCAGGGCCAAGUG
#2-2-24	ACUUGGCCAUGUUCGUUUUCGCCCCGUGGCUGCAGGGCCAAGUG
#3-2-3	ACUUGGCCAUCUUACAAUGUUUAGAAGGUCUGCAGGGCCAAGUG
#4-1-20	ACUUGGCCAUGUACAGAAUUCUCCCGGGUCUGCAGGGCCAAGUG
#5-2-15	ACUUGGCCAAUUACCGUAGUUUUUGUAGUCUGCAGGGCCAAGUG

Nucleotide sequences

Synthesized random oligo pool:

AGCAAGTTAAAATAAGGCTAGTCCGTTATCAACTTGGCCAnnnnnnnnnnnnnnnnnnnn

CTGCAGGGCCAAGTGGCACCGAGTCGGTGCTTTTTATCGA

sgRNA shuttle vector (the sgRNA-1.2 BsmBI scaffold): hU6-gLuc sgRNA-scaffold-1.2 BsmBI-scaffold-polyT:

GAGGGCCTATTTCCCATGATTCCTTCATATTTGCATATACGATACAAGGCTGTTAG
AGAGATAATTAGAATTAATTTGACTGTAAACACAAAGATATTAGTACAAAATACG
TGACGTAGAAAGTAATAATTTCTTGGGTAGTTTGCAGTTTTAAAATTATGTTTTAA
AATGGACTATCATATGCTTACCGTAACTTGAAAGTATTTCGATTTCTTGGCTTTAT
ATATCTTGTGGAAAGGACGAAACACCGATCTAGATACGACTCACTATGTTTAAGA
GCTAGAAATAGCAAGTTAAAATAAGGCTAGTCCGTTATCAACTTGGCCAGGAGA
CGGACGTCTCACTGCAGGGCCAAGTGGCACCGAGTCGGTGCTTTTT

sgRNA Vector inserted with N20 random aptamer: hU6-gLuc sgRNA-scaffold-1.2 N20-aptamer-scaffold-polyT:

GAGGGCCTATTTCCCATGATTCCTTCATATTTGCATATACGATACAAGGCTGTTAG
AGAGATAATTAGAATTAATTTGACTGTAAACACAAAGATATTAGTACAAAATACG
TGACGTAGAAAGTAATAATTTCTTGGGTAGTTTGCAGTTTTAAAATTATGTTTTAA
AATGGACTATCATATGCTTACCGTAACTTGAAAGTATTTCGATTTCTTGGCTTTAT
ATATCTTGTGGAAAGGACGAAACACCGATCTAGATACGACTCACTATGTTTAAGA
GCTAGAAATAGCAAGTTAAAATAAGGCTAGTCCGTTATCAACTTGGCCANNNNN
NNNNNNNNNNNNNNNCTGCAGGGCCAAGTGGCACCGAGTCGGTGCTTTTT

hU6-gLuc sgRNA-scaffold-1.2 BsmBI-scaffold-polyT-EF1a-NLS-linker-EcoRI-linker-VPH:

GAGGGCCTATTTCCCATGATTCCTTCATATTTGCATATACGATACAAGGCTGTTAG
AGAGATAATTAGAATTAATTTGACTGTAAACACAAAGATATTAGTACAAAATACG
TGACGTAGAAAGTAATAATTTCTTGGGTAGTTTGCAGTTTTAAAATTATGTTTTAA
AATGGACTATCATATGCTTACCGTAACTTGAAAGTATTTCGATTTCTTGGCTTTAT
ATATCTTGTGGAAAGGACGAAACACCGATCTAGATACGACTCACTATGTTTAAGA
GCTAGAAATAGCAAGTTAAAATAAGGCTAGTCCGTTATCAACTTGGCCAGGAGA
CGGACGTCTCACTGCAGGGCCAAGTGGCACCGAGTCGGTGCTTTTTATCGATATA
AGCTTTGCAAAGATGGATAAAGTTTTAAACAGAGAGGAATCTTTGCAGCTAATGG
ACCTTCTAGGTCTTGAAAGGAGTGGGAATTGGCTCCGGTGCCCGTCAGTGGGCAG
AGCGCACATCGCCCACAGTCCCCGAGAAGTTGGGGGGAGGGGTCGGCAATTGAA
CCGGTGCCTAGAGAAGGTGGCGCGGGGTAAACTGGGAAAGTGATGTCGTGTACT
GGCTCCGCCTTTTTCCCGAGGGTGGGGGAGAACCGTATATAAGTGCAGTAGTCGC
CGTGAACGTTCTTTTTCGCAACGGGTTTGCCGCCAGAACACAGGTAAGTGCCGTG
TGTGGTTCCCGCGGGCCTGGCCTCTTTACGGGTTATGGCCCTTGCGTGCCTTGAAT
TACTTCCACCTGGCTGCAGTACGTGATTCTTGATCCCGAGCTTCGGGTTGGAAGTG
GGTGGGAGAGTTCGAGGCCTTGCGCTTAAGGAGCCCCTTCGCCTCGTGCTTGAGT
TGAGGCCTGGCCTGGGCGCTGGGGCCGCCGCGTGCGAATCTGGTGGCACCTTCGC
GCCTGTCTCGCTGCTTTCGATAAGTCTCTAGCCATTTAAAATTTTTGATGACCTGC
TGCGACGCTTTTTTTCTGGCAAGATAGTCTTGTAAATGCGGGCCAAGATCTGCAC
ACTGGTATTTCGGTTTTTGGGGCCGCGGGCGGCGACGGGGCCCGTGCGTCCCAGC
GCACATGTTCGGCGAGGCGGGGCCTGCGAGCGCGGCCACCGAGAATCGGACGGG
GGTAGTCTCAAGCTGGCCGGCCTGCTCTGGTGCCTGGCCTCGCGCCGCCGTGTAT
CGCCCCGCCCTGGGCGGCAAGGCTGGCCCGGTCGGCACCAGTTGCGTGAGCGGA
AAGATGGCCGCTTCCCGGCCCTGCTGCAGGGAGCTCAAAATGGAGGACGCGGCG
CTCGGGAGAGCGGGCGGGTGAGTCACCCACACAAAGGAAAAGGGCCTTTCCGTC
CTCAGCCGTCGCTTCATGTGACTCCACGGAGTACCGGGCGCCGTCCAGGCACCTC
GATTAGTTCTCGAGCTTTTGGAGTACGTCGTCTTTAGGTTGGGGGGAGGGGTTTTA
TGCGATGGAGTTTCCCCACACTGAGTGGGTGGAGACTGAAGTTAGGCCAGCTTGG
CACTTGATGTAATTCTCCTTGGATCCACTAGTGGCTCTAGAGCCACCATGGGACCT
AAGAAAAAGAGGAAGGTGGCGGCCGCTGGTGGCTCTGGCGGTAGTGGTGGTGAA
TTCGGTGGCTCTGGCGGTAGTGGTGGTAGCGATCGCGGGTCGGATGCTTTAGACG
ATTTTGACTTAGATATGCTTGGTTCAGACGCGTTAGACGACTTCGACCTAGACAT
GTTAGGCTCAGATGCATTGGACGACTTCGATTTAGATATGTTGGGCTCCGATGCC
CTAGATGACTTTGATCTAGATATGCTAGGTAGTAGTGGGGGAGGAGGTGGAAGC

GCGATTAACAGCCAGTACCTGCCCGACACCGACGACCGGCACCGGATCGAGGAA
AAGCGGAAGCGGACCTACGAGACATTCAAGAGCATCATGAAGAAGTCCCCCTTC
AGCGGCCCCACCGACCCTAGACCTCCACCTAGAAGAATCGCCGTGCCCAGCAGAT
CCAGCGCCAGCGTGCCAAAACCTGCCCCCCAGCCTTACCCCTTCACCAGCAGCCT
GAGCACCATCAACTACGACGAGTTCCCTACCATGGTGTTCCCCAGCGGCCAGATC
TCTCAGGCCTCTGCTCTGGCTCCAGCCCCTCCTCAGGTGCTGCCTCAGGCTCCTGC
TCCTGCACCAGCTCCAGCCATGGTGTCTGCACTGGCTCAGGCACCAGCACCCGTG
CCTGTGCTGGCTCCTGGACCTCCACAGGCTGTGGCTCCACCAGCCCCTAAACCTA
CACAGGCCGGCGAGGGCACACTGTCTGAAGCTCTGCTGCAGCTGCAGTTCGACGA
CGAGGATCTGGGAGCCCTGCTGGGAAACAGCACCGATCCTGCCGTGTTCACCGAC
CTGGCCAGCGTGGACAACAGCGAGTTCCAGCAGCTGCTGAACCAGGGCATCCCT
GTGGCCCCTCACACCACCGAGCCCATGCTGATGGAATACCCCGAGGCCATCACCC
GGCTCGTGACAGGCGCTCAGAGGCCTCCTGATCCAGCTCCTGCCCCTCTGGGAGC
ACCAGGCCTGCCTAATGGACTGCTGTCTGGCGACGAGGACTTCAGCTCTATCGCC
GATATGGATTTCTCAGCCTTGCTGAGTGGGGGAGGAGGTGGAAGCGGCTTCAGCG
TGGACACCAGTGCCCTGCTGGACCTGTTCAGCCCCTCGGTGACCGTGCCCGACAT
GAGCCTGCCTGACCTTGACAGCAGCCTGGCCAGTATCCAAGAGCTCCTGTCTCCC
CAGGAGCCCCCCAGGCCTCCCGAGGCAGAGAACAGCAGCCCGGATTCAGGGAAG
CAGCTGGTGCACTACACAGCGCAGCCGCTGTTCCTGCTGGACCCCGGCTCCGTGG
ACACCGGGAGCAACGACCTGCCGGTGCTGTTTGAGCTGGGAGAGGGCTCCTACTT
CTCCGAAGGGGACGGCTTCGCCGAGGACCCCACCATCTCCCTGCTGACAGGCTCG
GAGCCTCCCAAAGCCAAGGACCCCACTGTCTCC

hU6-gLuc sgRNA-scaffold-1.2 BsmBI-scaffold-polyT-EF1a-NLS-linker-EcoRI-WT RBD-EcoRI-linker-VPH:

GAGGGCCTATTTCCCATGATTCCTTCATATTTGCATATACGATACAAGGCTGTTAG
AGAGATAATTAGAATTAATTTGACTGTAAACACAAAGATATTAGTACAAAATACG
TGACGTAGAAAGTAATAATTTCTTGGGTAGTTTGCAGTTTTAAAATTATGTTTTAA
AATGGACTATCATATGCTTACCGTAACTTGAAAGTATTTCGATTTCTTGGCTTTAT
ATATCTTGTGGAAAGGACGAAACACCGATCTAGATACGACTCACTATGTTTAAGA
GCTAGAAATAGCAAGTTAAAATAAGGCTAGTCCGTTATCAACTTGGCCAGGAGA
CGGACGTCTCACTGCAGGGCCAAGTGGCACCGAGTCGGTGCTTTTTATCGATATA
AGCTTTGCAAAGATGGATAAAGTTTTAAACAGAGAGGAATCTTTGCAGCTAATGG
ACCTTCTAGGTCTTGAAAGGAGTGGGAATTGGCTCCGGTGCCCGTCAGTGGGCAG
AGCGCACATCGCCCACAGTCCCCGAGAAGTTGGGGGGAGGGGTCGGCAATTGAA

CCGGTGCCTAGAGAAGGTGGCGCGGGGTAAACTGGGAAAGTGATGTCGTGTACT
GGCTCCGCCTTTTTCCCGAGGGTGGGGGAGAACCGTATATAAGTGCAGTAGTCGC
CGTGAACGTTCTTTTTCGCAACGGGTTTGCCGCCAGAACACAGGTAAGTGCCGTG
TGTGGTTCCCGCGGGCCTGGCCTCTTTACGGGTTATGGCCCTTGCGTGCCTTGAAT
TACTTCCACCTGGCTGCAGTACGTGATTCTTGATCCCGAGCTTCGGGTTGGAAGTG
GGTGGGAGAGTTCGAGGCCTTGCGCTTAAGGAGCCCCTTCGCCTCGTGCTTGAGT
TGAGGCCTGGCCTGGGCGCTGGGGCCGCCGCGTGCGAATCTGGTGGCACCTTCGC
GCCTGTCTCGCTGCTTTCGATAAGTCTCTAGCCATTTAAAATTTTTGATGACCTGC
TGCGACGCTTTTTTTCTGGCAAGATAGTCTTGTAAATGCGGGCCAAGATCTGCAC
ACTGGTATTTCGGTTTTTGGGGCCGCGGGCGGCGACGGGGCCCGTGCGTCCCAGC
GCACATGTTCGGCGAGGCGGGGCCTGCGAGCGCGGCCACCGAGAATCGGACGGG
GGTAGTCTCAAGCTGGCCGGCCTGCTCTGGTGCCTGGCCTCGCGCCGCCGTGTAT
CGCCCCGCCCTGGGCGGCAAGGCTGGCCCGGTCGGCACCAGTTGCGTGAGCGGA
AAGATGGCCGCTTCCCGGCCCTGCTGCAGGGAGCTCAAAATGGAGGACGCGGCG
CTCGGGAGAGCGGGCGGGTGAGTCACCCACACAAAGGAAAAGGGCCTTTCCGTC
CTCAGCCGTCGCTTCATGTGACTCCACGGAGTACCGGGCGCCGTCCAGGCACCTC
GATTAGTTCTCGAGCTTTTGGAGTACGTCGTCTTTAGGTTGGGGGGAGGGGTTTTA
TGCGATGGAGTTTCCCCACACTGAGTGGGTGGAGACTGAAGTTAGGCCAGCTTGG
CACTTGATGTAATTCTCCTTGGATCCACTAGTGGCTCTAGAGCCACCATGGGACCT
AAGAAAAAGAGGAAGGTGGCGGCCGCTGGTGGCTCTGGCGGTAGTGGTGGTGAA
TTCAGAGTCCAACCAACAGAATCTATTGTTAGATTTCCTAATATTACAAACTTGTG
CCCTTTTGGTGAAGTTTTTAACGCCACCAGATTTGCATCTGTTTATGCTTGGAACA
GGAAGAGAATCAGCAACTGTGTTGCTGATTATTCTGTCCTATATAATTCCGCATC
ATTTTCCACTTTTAAGTGTTATGGAGTGTCTCCTACTAAATTAAATGATCTCTGCT
TTACTAATGTCTATGCAGATTCATTTGTAATTAGAGGTGATGAAGTCAGACAAAT
CGCTCCAGGGCAAACTGGAAAGATTGCTGATTATAATTATAAATTACCAGATGAT
TTTACAGGCTGCGTTATAGCTTGGAATTCTAACAATCTTGATTCTAAGGTTGGTGG
TAATTATAATTACCTGTATAGATTGTTTAGGAAGTCTAATCTCAAACCTTTTGAGA
GAGATATTTCAACTGAAATCTATCAGGCCGGTAGCACACCTTGTAATGGTGTTGA
AGGTTTTAATTGTTACTTTCCTTTACAATCATATGGTTTCCAACCCACTAATGGTG
TTGGTTACCAACCATACAGAGTAGTAGTACTTTCTTTTGAACTTCTACATGCACCA
GCAACTGTTTGTGGACCTAAAAAGTCTACTAATTTGGTTAAAAACAAATGTGTCA
ATTTCGAATTCGGTGGCTCTGGCGGTAGTGGTGGTAGCGATCGCGGGTCGGATGC
TTTAGACGATTTTGACTTAGATATGCTTGGTTCAGACGCGTTAGACGACTTCGACC

TAGACATGTTAGGCTCAGATGCATTGGACGACTTCGATTTAGATATGTTGGGCTC
CGATGCCCTAGATGACTTTGATCTAGATATGCTAGGTAGTAGTGGGGGAGGAGGT
GGAAGCGCGATTAACAGCCAGTACCTGCCCGACACCGACGACCGGCACCGGATC
GAGGAAAAGCGGAAGCGGACCTACGAGACATTCAAGAGCATCATGAAGAAGTCC
CCCTTCAGCGGCCCCACCGACCCTAGACCTCCACCTAGAAGAATCGCCGTGCCCA
GCAGATCCAGCGCCAGCGTGCCAAAACCTGCCCCCCAGCCTTACCCCTTCACCAG
CAGCCTGAGCACCATCAACTACGACGAGTTCCCTACCATGGTGTTCCCCAGCGGC
CAGATCTCTCAGGCCTCTGCTCTGGCTCCAGCCCCTCCTCAGGTGCTGCCTCAGGC
TCCTGCTCCTGCACCAGCTCCAGCCATGGTGTCTGCACTGGCTCAGGCACCAGCA
CCCGTGCCTGTGCTGGCTCCTGGACCTCCACAGGCTGTGGCTCCACCAGCCCCTA
AACCTACACAGGCCGGCGAGGGCACACTGTCTGAAGCTCTGCTGCAGCTGCAGTT
CGACGACGAGGATCTGGGAGCCCTGCTGGGAAACAGCACCGATCCTGCCGTGTTC
ACCGACCTGGCCAGCGTGGACAACAGCGAGTTCCAGCAGCTGCTGAACCAGGGC
ATCCCTGTGGCCCCTCACACCACCGAGCCCATGCTGATGGAATACCCCGAGGCCA
TCACCCGGCTCGTGACAGGCGCTCAGAGGCCTCCTGATCCAGCTCCTGCCCCTCT
GGGAGCACCAGGCCTGCCTAATGGACTGCTGTCTGGCGACGAGGACTTCAGCTCT
ATCGCCGATATGGATTTCTCAGCCTTGCTGAGTGGGGGAGGAGGTGGAAGCGGCT
TCAGCGTGGACACCAGTGCCCTGCTGGACCTGTTCAGCCCCTCGGTGACCGTGCC
CGACATGAGCCTGCCTGACCTTGACAGCAGCCTGGCCAGTATCCAAGAGCTCCTG
TCTCCCCAGGAGCCCCCCAGGCCTCCCGAGGCAGAGAACAGCAGCCCGGATTCA
GGGAAGCAGCTGGTGCACTACACAGCGCAGCCGCTGTTCCTGCTGGACCCCGGCT
CCGTGGACACCGGGAGCAACGACCTGCCGGTGCTGTTTGAGCTGGGAGAGGGCT
CCTACTTCTCCGAAGGGGACGGCTTCGCCGAGGACCCCACCATCTCCCTGCTGAC
AGGCTCGGAGCCTCCCAAAGCCAAGGACCCCACTGTCTCC

Amino acid sequences

WT spike (GISAID accession ID: EPI_ISL_414631)

MFLLTTKRTMFVFLVLLPLVSSQCVNLTTRTQLPPAYTNSFTRGVYYPDKVFRSSVLH
STQDLFLPFFSNVTWFHAIHVSGTNGTKRFDNPVLPFNDGVYFASTEKSNIIRGWIFGT
TLDSKTQSLLIVNNATNVVIKVCEFQFCNDPFLGVYYHKNNKSWMESEFRVYSSANN
CTFEYVSQPFLMDLEGKQGNFKNLREFVFKNIDGYFKIYSKHTPINLVRDLPQGFSAL
EPLVDLPIGINITRFQTLLALHRSYLTPGDSSSGWTAGAAAYYVGYLQPRTFLLKYNE
NGTITDAVDCALDPLSETKCTLKSFTVEKGIYQTSNFRVQPTESIVRFPNITNLCPFGEV
FNATRFASVYAWNRKRISNCVADYSVLYNSASFSTFKCYGVSPTKLNDLCFTNVYAD
SFVIRGDEVRQIAPGQTGKIADYNYKLPDDFTGCVIAWNSNNLDSKVGGNYNYLYRL

FRKSNLKPFERDISTEIYQAGSTPCNGVEGFNCYFPLQSYGFQPTNGVGYQPYRVVVL
SFELLHAPATVCGPKKSTNLVKNKCVNFNFNGLTGTGVLTESNKKFLPFQQFGRDIA
DTTDAVRDPQTLEILDITPCSFGGVSVITPGTNTSNQVAVLYQGVNCTEVPVAIHADQ
LTPTWRVYSTGSNVFQTRAGCLIGAEHVNNSYECDIPIGAGICASYQTQTNSPRRARS
VASQSIIAYTMSLGAENSVAYSNNSIAIPTNFTISVTTEILPVSMTKTSVDCTMYICGDS
TECSNLLLQYGSFCTQLNRALTGIAVEQDKNTQEVFAQVKQIYKTPPIKDFGGFNFSQI
LPDPSKPSKRSFIEDLLFNKVTLADAGFIKQYGDCLGDIAARDLICAQKFNGLTVLPPL
LTDEMIAQYTSALLAGTITSGWTFGAGAALQIPFAMQMAYRFNGIGVTQNVLYENQ
KLIANQFNSAIGKIQDSLSSTASALGKLQDVVNQNAQALNTLVKQLSSNFGAISSVLN
DILSRLDKVEAEVQIDRLITGRLQSLQTYVTQQLIRAAEIRASANLAATKMSECVLGQ
SKRVDFCGKGYHLMSFPQSAPHGVVFLHVTYVPAQEKNFTTAPAICHDGKAHFPREG
VFVSNGTHWFVTQRNFYEPQIITTDNTFVSGNCDVVIGIVNNTVYDPLQPELDSFKEE
LDKYFKNHTSPDVDLGDISGINASVVNIQKEIDRLNEVAKNLNESLIDLQELGKYEQYI
KWPWYIWLGFIAGLIAIVMVTIMLCCMTSCCSCLKGCCSCGSCCKFDEDDSEPVLKG
VKLHYT

WT-RBD of spike (GISAID accession ID: EPI_ISL_414631)

RVQPTESIVRFPNITNLCPFGEVFNATRFASVYAWNRKRISNCVADYSVLYNSASFSTF
KCYGVSPTKLNDLCFTNVYADSFVIRGDEVRQIAPGQTGKIADYNYKLPDDFTGCVI
AWNSNNLDSKVGGNYNYLYRLFRKSNLKPFERDISTEIYQAGSTPCNGVEGFNCYFP
LQSYGFQPTNGVGYQPYRVVVLSFELLHAPATVCGPKKSTNLVKNKCVNF

Delta spike (GISAID accession ID: EPI_ISL_2356230)

MFLLTTKRTMFVFLVLLPLVSSQCVNLRTRTQLPPAYTNSFTRGVYYPDKVFRSSVLH
STQDLFLPFFSNVTWFHAIHVSGTNGTKRFDNPVLPFNDGVYFASIEKSNIIRGWIFGT
TLDSKTQSLLIVNNATNVVIKVCEFQFCNDPFLDVYYHKNNKSWMESGVYSSANNC
TFEYVSQPFLMDLEGKQGNFKNLREFVFKNIDGYFKIYSKHTPINLVRDLPQGFSALE
PLVDLPIGINITRFQTLLALHRSYLTPGDSSSGWTAGAAAYYVGYLQPRTFLLKYNEN
GTITDAVDCALDPLSETKCTLKSFTVEKGIYQTSNFRVQPTESIVRFPNITNLCPFGEVF
NATRFASVYAWNRKRISNCVADYSVLYNSASFSTFKCYGVSPTKLNDLCFTNVYADS
FVIRGDEVRQIAPGQTGKIADYNYKLPDDFTGCVIAWNSNNLDSKVGGNYNYRYRLF
RKSNLKPFERDISTEIYQAGSKPCNGVEGFNCYFPLQSYGFQPTNGVGYQPYRVVVLS
FELLHAPATVCGPKKSTNLVKNKCVNFNFNGLTGTGVLTESNKKFLPFQQFGRDIAD
TTDAVRDPQTLEILDITPCSFGGVSVITPGTNTSNQVAVLYQGVNCTEVPVAIHADQL
TPTWRVYSTGSNVFQTRAGCLIGAEHVNNSYECDIPIGAGICASYQTQTNSRRRARSV
ASQSIIAYTMSLGAENSVAYSNNSIAIPTNFTISVTTEILPVSMTKTSVDCTMYICGDST

ECSNLLLQYGSFCTQLNRALTGIAVEQDKNTQEVFAQVKQIYKTPPIKDFGGFNFSQIL
PDPSKPSKRSFIEDLLFNKVTLADAGFIKQYGDCLGDIAARDLICAQKFNGLTVLPPLL
TDEMIAQYTSALLAGTITSGWTFGAGAALQIPFAMQMAYRFNGIGVTQNVLYENQK
LIANQFNSAIGKIQDSLSSTASALGKLQNVVNQNAQALNTLVKQLSSNFGAISSVLNDI
LSRLDKVEAEVQIDRLITGRLQSLQTYVTQQLIRAAEIRASANLAATKMSECVLGQSK
RVDFCGKGYHLMSFPQSAPHGVVFLHVTYVPAQEKNFTTAPAICHDGKAHFPREGVF
VSNGTHWFVTQRNFYEPQIITTDNTFVSGNCDVVIGIVNNTVYDPLQPELDSFKEELD
KYFKNHTSPDVDLGDISGINASVVNIQKEIDRLNEVAKNLNESLIDLQELGKYEQYIK
WPWYIWLGFIAGLIAIVMVTIMLCCMTSCCSCLKGCCSCGSCCKFDEDDSEPVLKGV
KLHYT

Omicron BA.1 spike (GISAID accession ID: EPI_ISL_6825398)

MFLLTTKRTMFVFLVLLPLVSSQCVNLTTRTQLPPAYTNSFTRGVYYPDKVFRSSVLH
STQDLFLPFFSNVTWFHVISGTNGTKRFDNPVLPFNDGVYFASIEKSNIIRGWIFGTTL
DSKTQSLLIVNNATNVVIKVCEFQFCNDPFLDHKNNKSWMESEFRVYSSANNCTFEY
VSQPFLMDLEGKQGNFKNLREFVFKNIDGYFKIYSKHTPIIVREPEDLPQGFSALEPLV
DLPIGINITRFQTLLALHRSYLTPGDSSSGWTAGAAAYYVGYLQPRTFLLKYNENGTI
TDAVDCALDPLSETKCTLKSFTVEKGIYQTSNFRVQPTESIVRFPNITNLCPFDEVFNA
TRFASVYAWNRKRISNCVADYSVLYNLAPFFTFKCYGVSPTKLNDLCFTNVYADSFV
IRGDEVRQIAPGQTGNIADYNYKLPDDFTGCVIAWNSNKLDSKVSGNYNYLYRLFRK
SNLKPFERDISTEIYQAGNKPCNGVAGFNCYFPLRSYSFRPTYGVGHQPYRVVVLSFE
LLHAPATVCGPKKSTNLVKNKCVNFNFNGLKGTGVLTESNKKFLPFQQFGRDIADTT
DAVRDPQTLEILDITPCSFGGVSVITPGTNTSNQVAVLYQGVNCTEVPVAIHADQLTP
TWRVYSTGSNVFQTRAGCLIGAEYVNNSYECDIPIGAGICASYQTQTKSHRRARSVAS
QSIIAYTMSLGAENSVAYSNNSIAIPTNFTISVTTEILPVSMTKTSVDCTMYICGDSTEC
SNLLLQYGSFCTQLKRALTGIAVEQDKNTQEVFAQVKQIYKTPPIKYFGGFNFSQILPD
PSKPSKRSFIEDLLFNKVTLADAGFIKQYGDCLGDIAARDLICAQKFKGLTVLPPLLTD
EMIAQYTSALLAGTITSGWTFGAGAALQIPFAMQMAYRFNGIGVTQNVLYENQKLIA
NQFNSAIGKIQDSLSSTASALGKLQDVVNHNAQALNTLVKQLSSKFGAISSVLNDIFS
RLDKVEAEVQIDRLITGRLQSLQTYVTQQLIRAAEIRASANLAATKMSECVLGQSKRV
DFCGKGYHLMSFPQSAPHGVVFLHVTYVPAQEKNFTTAPAICHDGKAHFPREGVFVS
NGTHWFVTQRNFYEPQIITTDNTFVSGNCDVVIGIVNNTVYDPLQPELDSFKEELDKY
FKNHTSPDVDLGDISGINASVVNIQKEIDRLNEVAKNLNESLIDLQELGKYEQYIKWP
WYIWLGFIAGLIAIVMVTIMLCCMTSCCSCLKGCCSCGSCCKFDEDDSEPVLKGVKL
HYT

Omicron BA.2 spike (GISAID accession ID: EPI_ISL_8207301)

MFLLTTKRTMFVFLVLLPLVSSQCVNLITRTQSYTNSFTRGVYYPDKVFRSSVLHSTQ
DLFLPFFSNVTWFHAIHVSGTNGTKRFDNPVLPFNDGVYFASTEKSNIIRGWIFGTTLD
SKTQSLLIVNNATNVVIKVCEFQFCNDPFLDVYYHKNNKSWMESEFRVYSSANNCTF
EYVSQPFLMDLEGKQGNFKNLREFVFKNIDGYFKIYSKHTPINLGRDLPQGFSALEPL
VDLPIGINITRFQTLLALHRSYLTPGDSSSGWTAGAAAYYVGYLQPRTFLLKYNENGT
ITDAVDCALDPLSETKCTLKSFTVEKGIYQTSNFRVQPTESIVRFPNITNLCPFDEVFNA
TRFASVYAWNRKRISNCVADYSVLYNFAPFFAFKCYGVSPTKLNDLCFTNVYADSFV
IRGNEVSQIAPGQTGNIADYNYKLPDDFTGCVIAWNSNNLDSKVGGNYNYLYRLFRK
SNLKPFERDISTEIYQAGNKPCNGVAGFNCYFPLRSYGFRPTYGVGHQPYRVVVLSFE
LLHAPATVCGPKKSTNLVKNKCVNFNFNGLTGTGVLTESNKKFLPFQQFGRDIADTT
DAVRDPQTLEILDITPCSFGGVSVITPGTNTSNQVAVLYQGVNCTEVPVAIHADQLTP
TWRVYSTGSNVFQTRAGCLIGAEYVNNSYECDIPIGAGICASYQTQTKSHRRARSVAS
QSIIAYTMSLGAENSVAYSNNSIAIPTNFTISVTTEILPVSMTKTSVDCTMYICGDSTEC
SNLLLQYGSFCTQLKRALTGIAVEQDKNTQEVFAQVKQIYKTPPIKYFGGFNFSQILPD
PSKPSKRSFIEDLLFNKVTLADAGFIKQYGDCLGDIAARDLICAQKFNGLTVLPPLLTD
EMIAQYTSALLAGTITSGWTFGAGAALQIPFAMQMAYRFNGIGVTQNVLYENQKLIA
NQFNSAIGKIQDSLSSTASALGKLQDVVNHNAQALNTLVKQLSSKFGAISSVLNDILS
RLDKVEAEVQIDRLITGRLQSLQTYVTQQLIRAAEIRASANLAATKMSECVLGQSKRV
DFCGKGYHLMSFPQSAPHGVVFLHVTYVPAQEKNFTTAPAICHDGKAHFPREGVFVS
NGTHWFVTQRNFYEPQIITTDNTFVSGNCDVVIGIVNNTVYDPLQPELDSFKEELDKY
FKNHTSPDVDLGDISGINASVVNIQKEIDRLNEVAKNLNESLIDLQELGKYEQYIKWP
WYIWLGFIAGLIAIVMVTIMLCCMTSCCSCLKGCCSCGSCCKFDEDDSEPVLKGVKL
HYT
